# A Single Sphingomyelin Species Promotes Exosomal Release of Endoglin into the Maternal Circulation in Preeclampsia

**DOI:** 10.1038/s41598-017-12491-4

**Published:** 2017-09-22

**Authors:** Leonardo Ermini, Jonathan Ausman, Megan Melland-Smith, Behzad Yeganeh, Alessandro Rolfo, Michael L. Litvack, Tullia Todros, Michelle Letarte, Martin Post, Isabella Caniggia

**Affiliations:** 1 Program in Translational Medicine, Hospital for Sick Children, Toronto, Ontario M5G 1X8 Canada; 2 Program in Molecular Medicine, Hospital for Sick Children, Toronto, Ontario M5G 1X8 Canada; 3 The Lunenfeld-Tanenbaum Research Institute, Mount Sinai Hospital, Toronto, Ontario M5G 1X5 Canada; 4 Institute of Medical Sciences, University of Toronto, Toronto, Ontario M5S 1A8 Canada; 5 Department of Obstetrics and Gynecology, University of Toronto, Toronto, Ontario M5S 1A8 Canada; 6 Department of Physiology, University of Toronto, Toronto, Ontario M5S 1A8 Canada; 7 Department of Laboratory Medicine and Pathobiology, University of Toronto, Toronto, Ontario M5S 1A8 Canada; 8 Department of Immunology, University of Toronto, Toronto, Ontario M5S 1A8 Canada; 9 Department of Obstetrics and Gynecology, University of Turin, Turin, 10126 Italy

## Abstract

Preeclampsia (PE), an hypertensive disorder of pregnancy, exhibits increased circulating levels of a short form of the auxillary TGF-beta (TGFB) receptor endoglin (sENG). Until now, its release and functionality in PE remains poorly understood. Here we show that ENG selectively interacts with sphingomyelin(SM)-18:0 which promotes its clustering with metalloproteinase 14 (MMP14) in SM-18:0 enriched lipid rafts of the apical syncytial membranes from PE placenta where ENG is cleaved by MMP14 into sENG. The SM-18:0 enriched lipid rafts also contain type 1 and 2 TGFB receptors (TGFBR1 and TGFBR2), but not soluble fms-like tyrosine kinase 1 (sFLT1), another protein secreted in excess in the circulation of women with PE. The truncated ENG is then released into the maternal circulation *via* SM-18:0 enriched exosomes together with TGFBR1 and 2. Such an exosomal TGFB receptor complex could be functionally active and block the vascular effects of TGFB in the circulation of PE women.

## Introduction

Preeclampsia (PE) complicates 5–7% of all pregnancies and is a major cause of maternal and perinatal morbidity and mortality worldwide. Aberrant expression of angiogenic modulators is important in the pathogenesis of preeclampsia, and their utility as biomarkers has been established^1,2^. Studies have shown that maternal circulating levels of placenta-derived anti-angiogenic proteins such as circulating endoglin (ENG) and soluble fms-like tyrosine kinase 1 receptor (sFLT1) are significantly higher in pregnancies complicated by preeclampsia than in normotensive control and pure intrauterine growth restricted (IUGR) pregnancies^3–5^. Soluble FLT1 is a splice variant of vascular endothelial growth factor (VEGF) receptor 1 (VEGFR1 or FLT1) that lacks a transmembrane domain (TMD) and captures circulating VEGF and placental growth factor (PIGF), thereby inducing a systemic endothelial dysfunction^6^. A shortened form of ENG (sENG) is produced by proteolytic cleavage of transforming growth factor beta (TGFB) co-receptor ENG^7^. The presence of sENG in the circulation interferes with TGFBsignaling, thereby affecting vascular homeostasis and inducing hypertension *in vivo* ^3^. The expression of sFLT1 and cleavage of full-length ENG into sENG are increased in preeclamptic placentae^8–12^, but the precise mechanism(s) responsible for processing and shedding of these anti-angiogenic factors by the syncytiotrophoblast layer and their mode of transport in the maternal circulation remains elusive.

Sphingolipids are a class of bioactive lipids containing a sphingoid backbone that include sphingosine, sphingosine-1-phosphate, ceramides, and sphingomyelins (SM). Sphingolipid levels (particularly SM) are increased during the last trimester of pregnancy in placental tissues and fetal membranes of several species, including humans^13^. Recently, we demonstrated that PE and IUGR are characterized by unique, yet diverse, altered sphingolipid profiles^14,15^. Sphingolipids not only participate in various signalling pathways, they also are important membrane constituents, and their molecular diversity influences the physicochemical nature of the cell membrane. SMs are dominant sphingolipids in membranes of mammalian cells, specifically of the plasma membrane, the endocytic recycling compartment, and the *trans*-Golgi network^16^. Sphingolipid-cholesterol interactions lead to the formation of membrane subdomains such as lipid rafts and caveolae^17^. The structure and function of lipid raft domains is critically dependent on their lipid and protein composition. Membrane proteins are sequestered in these sphingolipid-enriched subdomains by specific interactions between TMDs and sphingolipids^18^. The gradient of sphingolipids across organelles of the secretory pathway is thought to be responsible for sorting of plasma membrane proteins^19^. Recently, sphingolipid-binding domains (SBDs) have been identified on soluble and membrane-bound proteins. For example, p24, a component of the HIV particle capsid, contains a SBD in its TMD and this SBD motif does not only bind to SM, but is selective for a single molecular SM species (SM-18:0)^20^. The specific interaction of p24 with SM-18:0 allosterically modulates the oligomeric state of the protein, thereby altering its function in coat protein complex protein I (COPI) vesicle biogenesis. Thus, sphingolipids may act as ‘vehicle chaperones’ by creating a distinctive physical milieu around ‘protein customers’. Whether particular sphingolipids target and interact with specific placental membrane proteins such as ENG and FLT1 is unknown.

In the present study, we examined the interaction of SM species with ENG and sFLT1 in membrane subdomains of placentae from normotensive and preeclamptic pregnancies. We found an altered distribution of sphingomyelin species, in particular SM-18:0, in apical (syncytial) membrane lipid rafts from PE placentae that promoted clustering of ENG with MMP14 and cleavage into a shortened ENG. The truncated ENG, but not sFLT1, was subsequently found in placental-derived SM-18:0 enriched exosomes in plasma of preeclamptic mothers, suggesting that sENG is shed by the syncytium into the maternal blood *via* exosomes while sFLT1 is directly released into circulation.

## Results

### Placental and circulating sphingomyelin levels are increased in preeclampsia

**Figure 1:**
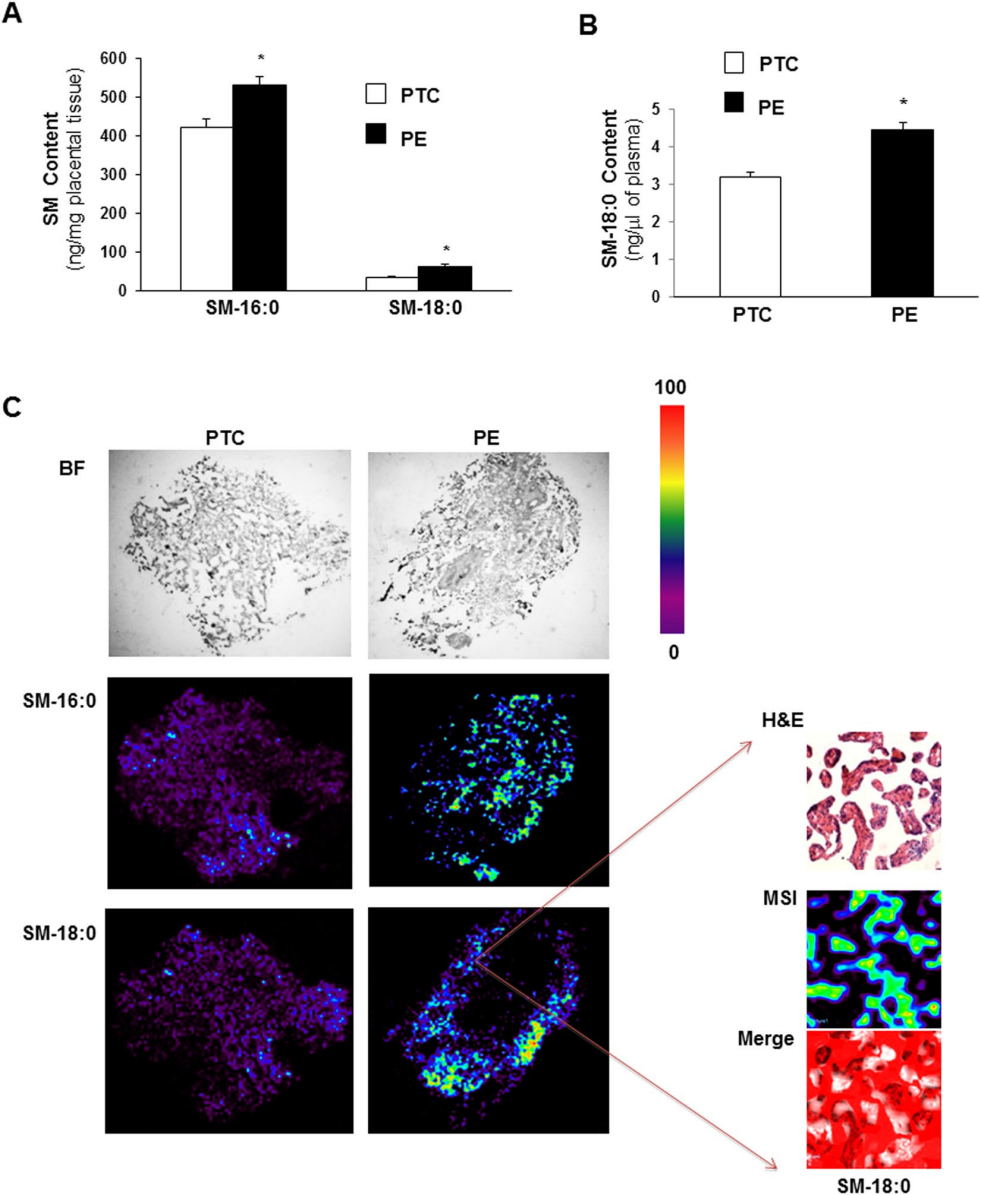
Sphingomyelin levels are increased in preeclamptic placentae. (**A**) Sphingomyelin (SM) levels measured by LC-MS/MS in placental tissue from preeclamptic (PE) women compared to normotensive age-matched preterm controls (PTC). SM numbers indicate fatty acid chain length on D-erythro-sphingosylphosphorylcholine backbone (PE, n = 45; PTC, n = 40 different placentae; *p < 0.05). (**B**) N-stearoyl sphingomyelin (SM-18:0) levels measured by LC-MS/MS in plasma from PE *vs* PTC women (PE, n = 10; TC, n = 10 separate samples; *p < 0.05). Data are presented as mean ± s.e.m. Significance was determined using an unpaired two-sided t-test. (**C**-left panels) Representative MALDI-MS images of SM-16:0 and SM-18:0 distributions in PE and PTC placental sections. Intensities of the ions based on the intensity scale provided. All imaging experiments were repeated with tissues obtained from 6 different PE and PTC placentae. (**C**-right panels) H&E and MALDI-MS images of SM-18:0 in PE placental terminal villi (magnification: 40x). White pseudocolor indicates overlap between MSI SM-18:0 and H&E stained terminal villi. Red is chosen as pseudocolor background.

Sphingolipidomic analysis revealed significant increases in N-palmitoyl (SM-16:0 (d18:1/16:0)) and N-stearoyl (SM-18:0 (d18:1/18:0)) sphingomyelin levels in PE placentae compared to age-matched preterm controls (PTC) (Fig. 1A). SM-18:0, but not SM-16:0, content was also increased in maternal plasma of PE *vs* PTC pregnancies (Fig. 1B). Matrix-Assisted Laser Desorption Ionization (MALDI)-MSI was employed to visualize the spatial distribution of SM-16:0 and SM-18:0 in PE and PTC placental tissues. MALDI-MSI confirmed elevated levels of both SM species in PE *vs* PTC placenta and localized their increase as illustrated for SM18:0 primarily to the terminal villi of PE placentae (Fig. 1C).

### Endoglin interacts with SM-18:0 in PE placentae

**Figure 2:**
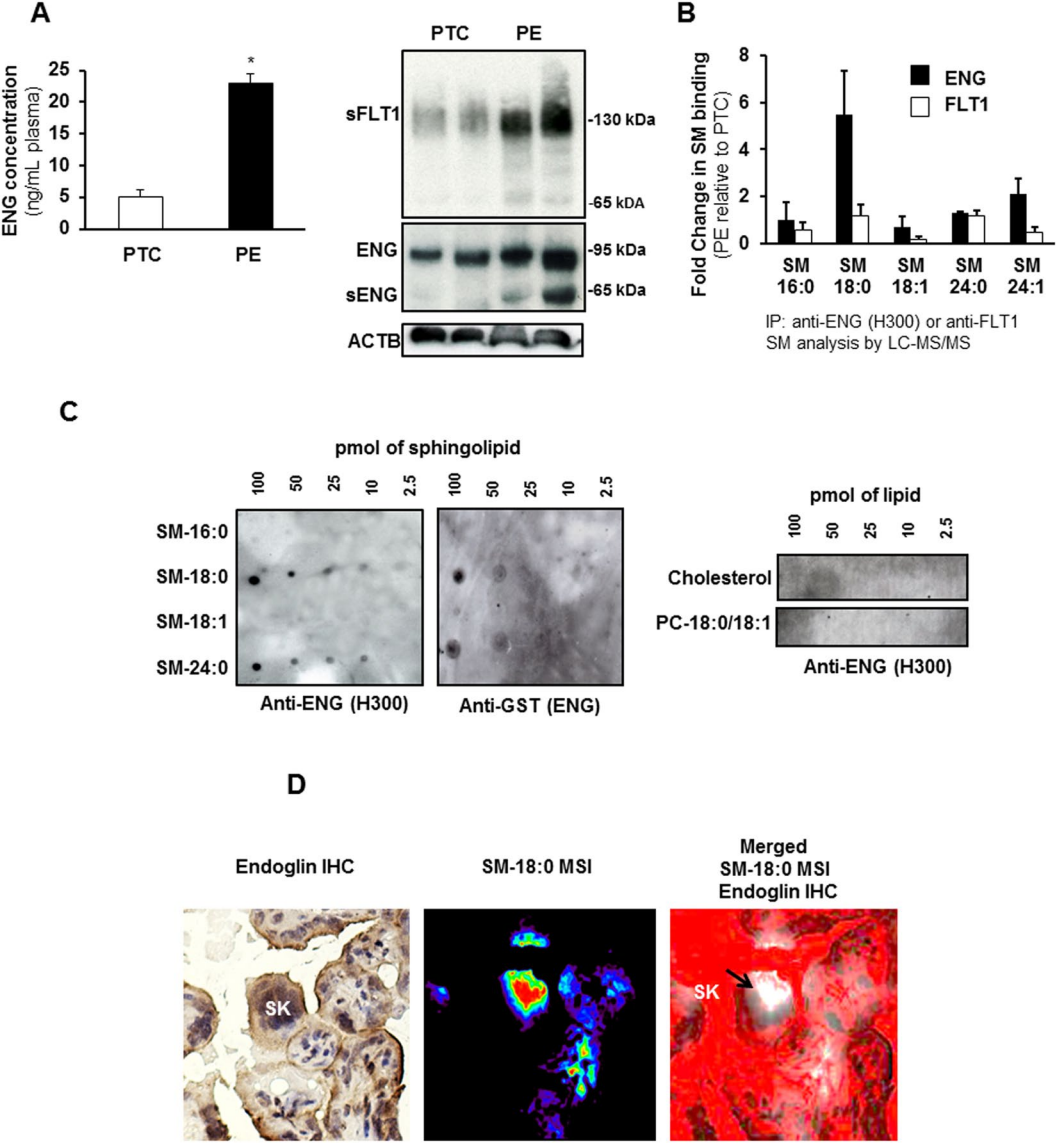
Endoglin associates with SM-18:0 in preeclamptic placenta. (**A**-left panel) ENG content (mean ± s.e.m.) measured by ELISA in plasma from preeclamptic (PE) women compared to normotensive age-matched preterm (PTC) controls (PE, n = 10 separate samples; PTC, n = 10 separate samples; *p < 0.05 by unpaired two-sided t-test). (**A**-right panel) Expression of ENG, sENG and sFLT1 in PE and PTC placentae (n = 2 placentae per group) as assessed by Western blotting. Experiment was repeated twice with similar results using different placentae. The images are cropped for clarity purposes. The full-length blots of ENG and ACTB are presented in Supplementary Fig. 6. (**B**) Sphingolipid analysis of ENG and FLT1 immunoprecipitates of PE and PTC placental lysates. Data (mean ± s.e.m.) are expressed as fold change in the amount of sphingomyelin (SM) associated with ENG or FLT1 in PE *vs* PTC placentae (PE, n = 3; PTC, n = 3 different placentae). SM numbers indicate fatty acid chain length on D-erythro-sphingosylphosphorylcholine backbone. (**C**) Interaction of GST-conjugated full-length ENG with several lipid species as assessed by protein overlay assay. Assay was repeated three times with similar results. (**D**) Spatial localization of ENG and SM-18:0 in PE placenta using MALDI-IMS and ENG immunohistochemistry (magnification: 40 X). Molecular distribution of SM-18:0 (middle panel) merged with ENG (brown immunoreactivity in left panel) is shown in right panel. White pseudocolor indicates MSI SM-18:0. Red is chosen as pseudocolor background. SK, syncytial knots, PC, phosphatidylcholine.

Although circulating ENG is elevated in preeclampsia (Fig. 2A-left panel) and both full-length and shortened ENG are increased in placental tissue (Fig. 2A-right panel) of women with preeclampsia, the formation and mode of release of sENG by the syncytium into the maternal circulation is poorly understood. Emerging evidence suggests that sphingolipid-protein interactions are important for various cellular processes^20,21^. Hence, we investigated the association of ENG (and FLT1) with sphingolipids. PTC and PE tissue lysates were immunoprecipitated with antibodies to ENG or FLT1 and the immunoprecipitates (IPs) were subjected to sphingolipid analysis using LC-MS/MS. There was a 6-fold enrichment of SM-18:0 in the ENG-IPs of PE compared to PTC placentae (Fig. 2B). No enrichment in any other SM (Fig. 2B) or ceramide (data not shown) species was found. The enrichment was significantly greater than the 2-fold increase in SM-18:0 content in PE placentae (Fig. 1A). The association of ENG with SM-18:0 was unique as IPs of the same tissue lysates with anti-FLT1 did not exhibit any sphingolipid enrichment (Fig. 2B). A Protein Lipid Overlay (PLO) assay confirmed the interaction of full-length ENG with SM-18:0 (Fig. 2C). The *in vitro* binding assay revealed also an interaction between ENG and SM-24:0 while no ENG binding with SM-16:0, SM-18:1, PC-18:0/18:1 and cholesterol was observed (Fig. 2C). Next, we investigated the spatial localization of ENG with SM-18:0 in PE placentae by MALDI-MSI and immunohistochemistry. Cryo-sections analyzed by MALDI-MSI for SM-18:0 were stained with an antibody to ENG after removal of the MALDI matrix with methanol, and the immunohistochemical images were then merged with the MALDI-MSI images. Positive ENG immunoreactivity was noted in areas of the syncytiotrophoblast layer where SM-18:0 was also present. In particular, we noted a strong overlap at level of the syncytial knots of the syncytiotrophoblast layer (Fig. 2D).

### SM-18:0 is increased in apical membrane lipid rafts of PE placentae

**Figure 3:**
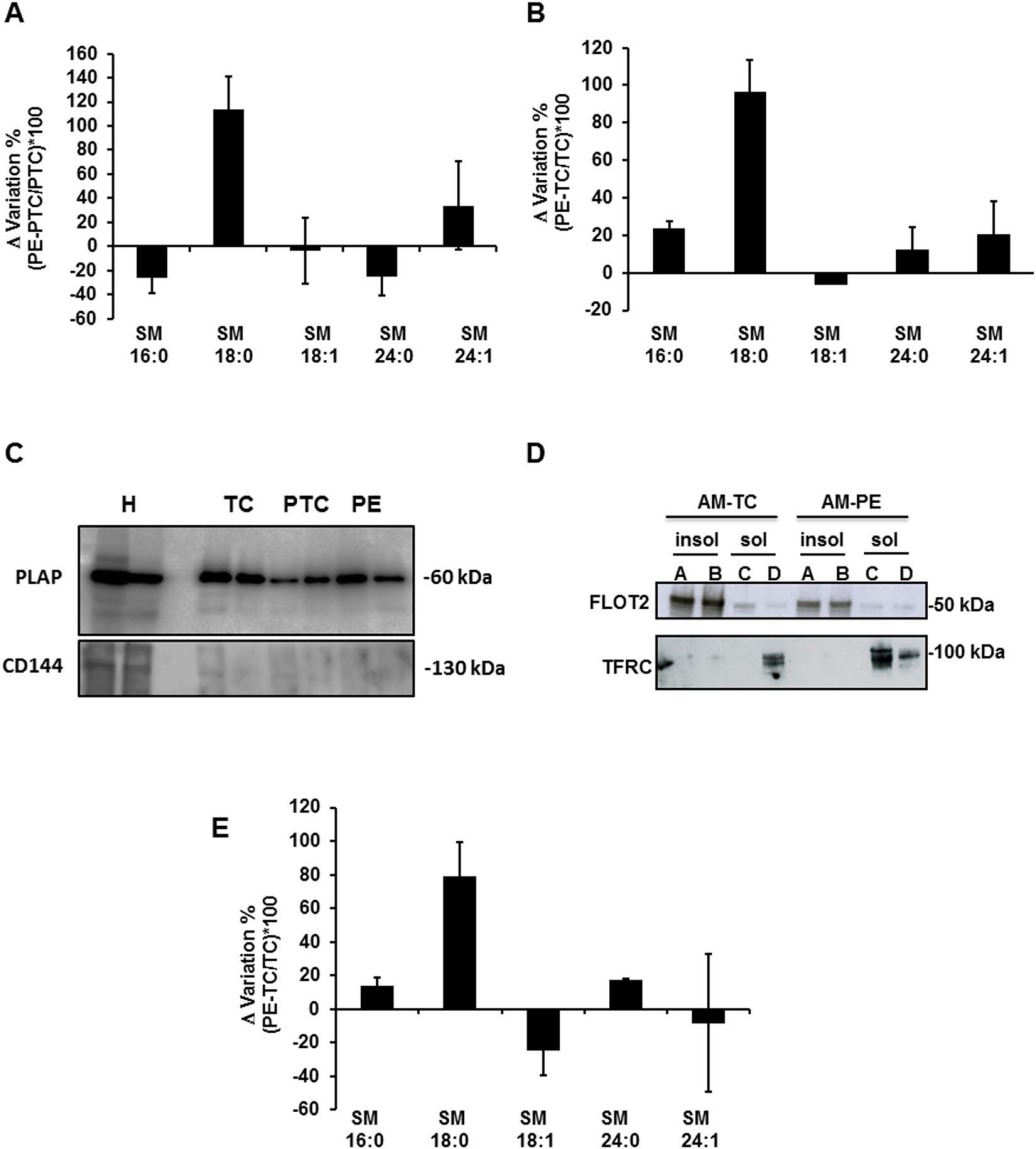
SM-18:0 content is increased in lipid rafts of preeclamptic membranes. (**A**,**B**) Alteration of SM species in detergent-resistant membrane (DRM) fractions of PE *vs* PTC (**A**) and PE *vs* TC (**B**) placenta. SM alteration (mean ± s.e.m.) is expressed as Δ variation (PE-TC/TC*100 or PE-PTC/PTC*100) (PE, n = 10; PTC, n = 6; TC, n = 4 different placentae). (**C**) Immunoblotting for PLAP and CD144 (VE-Cadherin) in apical (syncytial) membrane enriched fractions of TC, PTC and PE placentae. H: TC whole tissue homogenate. The images are cropped for clarity purposes. The full-length blots are presented in Supplementary Fig. 7. (**D**) Distribution of flottilin-2 (FLOT2) and transferrin receptor (TFRC) in detergent insoluble (**A**,**B**) and soluble (**C**,**D**) fractions of apical (syncytial) membranes (AM) of TC and PE placentae. Experiment was repeated twice with similar results using different placentae. The images are cropped for clarity purposes. The full-length blots are presented in Supplementary Fig. 7 (**E**) Alteration of SM species in DRMs of PE *vs* TC apical (syncytial) membranes. SM alteration (mean ± s.e.m.) is expressed as Δ variation (PE-TC/TC*100) (PE, n = 3; TC, n = 3 different placentae; lipid analysis carried out in duplicate).

Since sphingomyelins are essential elements of lipid rafts and caveolae we examined whether the aforementioned association of ENG with SM-18:0 in PE placentae occurs in these lipid microdomains. The isolation of lipid rafts is commonly performed using nonionic mild detergents like Triton-X100^22–25^. Detergent resistant membranes (DRMs) were isolated from term control (TC), PTC and PE placentae using sucrose density gradient centrifugation (see supplementary Results and Supplementary Fig. 1). The DRM-containing fractions of PTC and PE placentae were then analyzed by tandem mass spectrometry for cholesterol and sphingomyelin content. No significant differences in total amount of cholesterol (PE: 1.35 ± 0.25 *vs* PTC: 1.62 ± 0.52 *vs* TC: 1.29 ± 0.56 ng/μg protein and sphingomyelins PE: 709.01 ± 88.19 *vs* PTC: 785.26 ± 122.18 *vs* TC: 448.01 ± 67.79 ng/μg cholesterol; PTC: n = 6, PE: n = 6, TC: n = 4 different placentae) were noted between DRMs from PE *vs* PTC and TC placental membranes. However, the distribution of SM species with various fatty acyl lengths was altered (Fig. 3A). In particular, the amount of SM-18:0 was significantly increased in DRMs of PE *vs* PTC and TC membranes (Fig. 3A,B and Supplementary Table 1A). Since the syncytium is in direct contact with the maternal blood, we isolated the apical (syncytial) microvillous membranes of PE, PTC and TC placentae using a well-established and validated protocol^26,27^. We confirmed the purity of the isolated apical microvillous membranes by blotting for placental alkaline phosphatase (PLAP) (Fig. 3C), a syncytiotrophoblast microvillous membrane marker that is absent from other cell membranes in the human placenta^27^. In addition, we evaluated any contamination with endothelial cell membranes by blotting for CD144 (VE-Cadherin), an endothelial apical membrane marker, which turned out negative (Fig. 3C). We then isolated the lipid rafts from the apical membranes. The apical DRMs (insoluble fractions A, B) contained the lipid raft marker flottilin-2^24^ while the transferrin receptor, a known non-lipid raft protein^28^, localized to detergent soluble membrane fractions (Fig. 3D). There is an ongoing debate in the placenta field regarding proper controls for preeclamptic placentae. Therefore, we compared the distribution of sphingolipids in lipid rafts of apical (syncytial) membranes from TC placentae (from healthy pregnancies at term obtained from Caesarean sections) *vs* PTC placentae (from women experiencing preterm labour following a variety of causes including idiopathic preterm labour) and observed no significant change in sphingolipid composition (Supplementary Fig. 2A). Based on these findings we choose TC as controls for all subsequent lipid raft analysis. The total cholesterol (PE: 1.12 ± 0.12 *vs* TC: 1.10 ± 0.17 μg/μg protein) and sphingomyelin (PE: 629.53 ± 35.37 *vs* TC: 579.34 ± 33.48 ng/μg cholesterol, n = 3 placentae per group) content was similar in DRMs from apical PE and TC microvillous membranes, but apical PE-DRMs were significantly enriched in SM-18:0 compared to those from TC-DRMs (Fig. 3E and Supplementary Table 1B).

### Truncated endoglin is present in SM-18:0-enriched membrane lipid rafts of PE placentae

**Figure 4:**
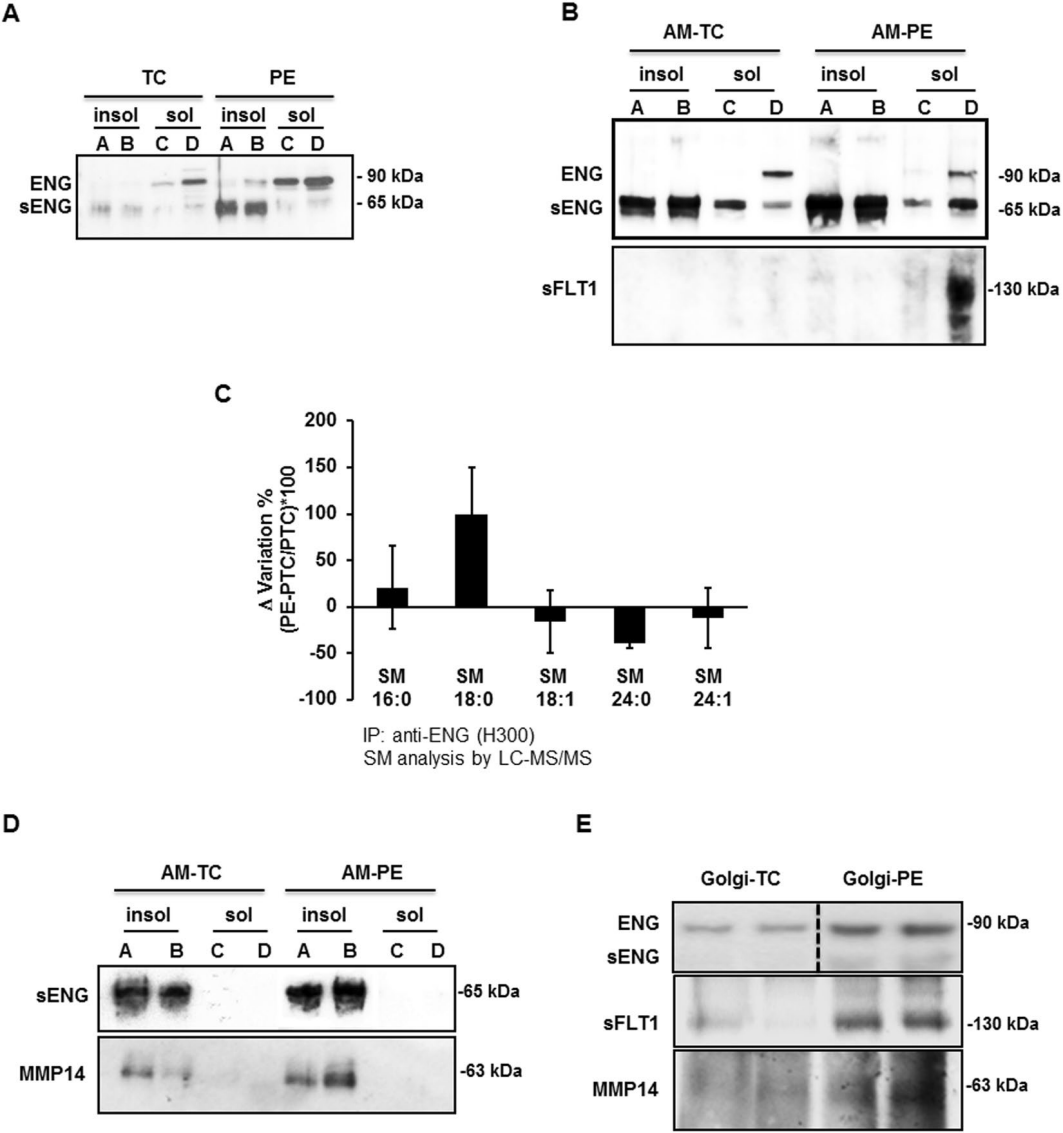
Truncated endoglin is present in SM18:0-enriched lipid rafts of apical (syncytial) membranes from PE placenta. (**A**,**B**) Distribution of ENG proteins and sFLT1 between detergent soluble and insoluble membrane fractions of PE and TC placenta. ENGs and sFLT1 distribution was assessed by immunoblotting after SDS-PAGE of detergent soluble and insoluble fractions of (**A**) total and (**B**-*upper panel:* ENG; **B**-*lower panel*: sFLT1) apical (syncytial) membranes (AM) of PE and TC placentae. Experiments were repeated two times with similar results using different placentae. The images are cropped for clarity purposes. The full-length blots are presented in Supplementary Fig. 8. (**C**) SM alterations in ENG immunoprecipitates of detergent insoluble membrane fractions of PE *vs* PTC placenta. The SM alteration is expressed as Δ variation (PE-PTC/PTC*100) (PE, n = 4, PTC, n = 4 different placentae). Data are presented as mean ± s.e.m. (**D)** Distribution of MMP14 and truncated ENG between detergent soluble and insoluble fractions of apical (syncytial) membranes (AM) from PE and TC placentae. Experiments were repeated three times with similar results using different placentae. The images are cropped for clarity purposes. The full-length blots are presented in Supplementary Fig. 8. (**E**) Immunoblots for ENG, sFLT1 and MMP14 in isolated PE and TC Golgi stacks (n = 2 different placentae per group). The images are cropped for clarity purposes. The full-length blots are presented in Supplementary Fig. 8. Experiments were repeated two times with similar results using different placentae.

As PE placentae exhibited an increased association between SM-18:0 and ENG, we reasoned those PE-DRMs containing high levels of SM-18:0 could also be enriched in ENG. The detergent insoluble and soluble membrane fractions isolated from TC and PE placentae were subjected to SDS-PAGE under reducing (Fig. 4A) and non-reducing (Supplementary Fig. 2B) conditions followed by immunoblotting with ENG (H300) antibody. Under reducing conditions, the H300 antibody recognized the reported full-length (≈90 kDa) ENG as well as the shortened (≈65 kDa) ENG (sENG; Fig. 4A). Intriguingly, DRMs (insoluble fractions A and B) of PE membranes were enriched in sENG compared to DRMs of TC membranes (Fig. 4A) while full-length ENG accumulated in the detergent soluble membrane fractions C and D. The distribution of both ENG proteins was similar in detergent soluble and insoluble fractions of PTC and TC membranes (data not shown). H300 antibody also identified the full length and shortened ENG under non-reducing conditions confirming the enrichment in monomeric sENG in PE *vs* TC DRMs (Supplementary Fig. 2B). Analysis of detergent soluble and insoluble fractions of apical (syncytial) microvillous membranes substantiated the enrichment of the truncated ENG in DRMs of PE *vs* TC apical membranes (Fig. 4B and Supplementary Fig. 2C). The presence of sENG in the apical (syncytial) DRMs was further corroborated by immunoprecipitation of the detergent insoluble fractions of apical membranes with P4A4 monoclonal antibody followed by immunoblotting with H300 antibody (Supplementary Fig. 2D). The P4A4 antibody recognizes an epitope in the extracellular domain of ENG^29^. By contrast, soluble FLT1 was solely present in the detergent soluble fractions of both total (Supplementary Fig. 2E) and apical (Fig. 4B) PE membranes. To determine whether sENG in DRMs of PE membranes associated with SM-18:0, we immunoprecipitated sENG from PE and PTC DRMs with H300 antibody and subjected the IPs to sphingolipidomic analysis. An increased association of the shortened ENG with SM-18:0 in PE *vs* PTC DRMs was noted (Fig. 4C). In contrast, full-length ENG in the soluble (non-raft) membrane compartment did not associate with any SM (Supplementary Fig. 2F). Subsequent *in vitro* PLO assays using a HIS-conjugated peptide encompassing the extracellular domain of ENG (Met_1_-Gly_586_) confirmed a direct interaction of this truncated ENG with SM-18:0 although the peptide bound more profoundly to SM-24:0 (Supplementary Fig. 3A).

### MMP14 is present in SM18:0-enriched membrane lipid rafts of PE placentae

Various membrane-type metalloproteases localize to the syncytiotrophoblast but only MMP14 is thought to be responsible for endoglin cleavage in the placenta^7,30,31^. Immunoblotting of detergent insoluble and soluble membrane fractions showed that both MMP14 and sENG are present in the DRMs of apical (syncytial) microvillous membranes (Fig. 4D, upper panels). Sphingolipidomic analysis of MMP14 immunoprecipitates showed a strikingly increased association of MMP14 with SM-18:0 in apical PE *vs* TC DRMs (Supplementary Fig. 3B), similar to that seen with the shortened ENG (Fig. 4C). A co-immunoprecipitation assay verified the joint presence of MMP14 and ENG within the apical PE DRMs (Supplementary Fig. 3C). To determine whether the cleavage of full-length ENG by MMP14 occurred at the level of the plasma membrane, fractions enriched with Golgi stacks were isolated from PE and TC placentae (Supplementary Fig. 4A). Subsequent immunoblotting with H300 antibody showed full-length ENG being present in the isolated Golgi fractions of PE and TC placentae while shortened ENG was barely discernible (Fig. 4E, upper panels). In contrast, soluble FLT1 was present in the Golgi vesicles of PE placenta, in agreement with it being expressed as a splice variant of FLT1^32^. MMP14 was also present in the PE Golgi stacks (Fig. 4E, upper panels) and associated with full-length ENG (Supplementary Fig. 4C). Interestingly, Golgi stacks from PE placental tissue were significantly enriched in SM-18:0 when compared to Golgi vesicles from TC placenta (Supplementary Fig. 4B and Supplementary Table 2) and both full-length ENG and MMP14 associated with SM-18:0 in the PE Golgi vesicles (Supplementary Fig. 4D,E).

### Low oxygen increases SM-18:0 in lipid rafts

**Figure 5:**
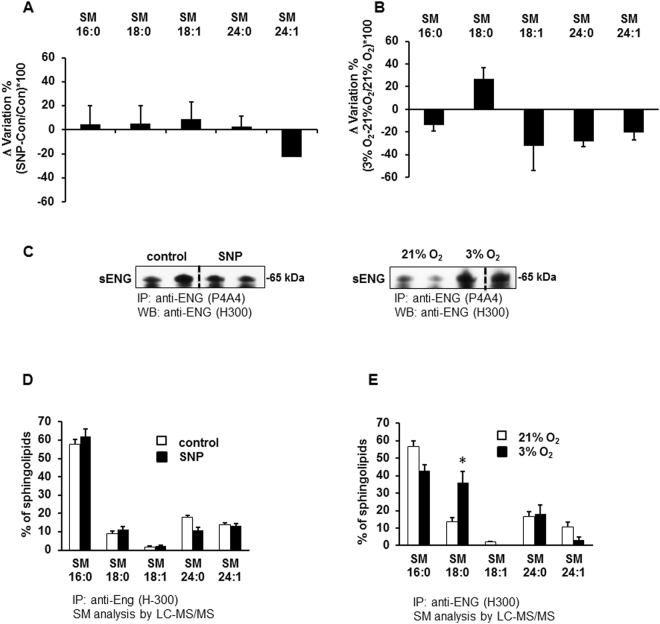
Low oxygen increases SM-18:0 content in lipid rafts and SM18:0 association with ENG. Sphingomyelin changes in the DRMs of first trimester placental villous explants treated (**A**) with or without 5 mM SNP (n = 6 explants per treatment) or (**B**) cultured under ambient (21%) or 3% O_2_ (n = 9 explants per treatment). Changes in SM species (mean ± s.e.m.) are expressed as Δ variation (SNP-control/control*100 or 3% O_2_-21% O_2_/21% O_2_*100). (**C**) Expression of the shortened endoglin (sENG) in DRMs from SNP-treated and 3%O_2_-exposed explants. The images are cropped for clarity purposes. The full-length blots are presented in Supplementary Fig. 9. (**D**,**E**) Sphingomyelins in anti-ENG precipitated DRMs from explants exposed to SNP (**D**) or 3%O_2_ (**E**). The distribution of SM species (mean ± s.e.m.) is expressed as percentage of total SM amount (SNP-treated explants, n = 6; O_2_-exposed explants, n = 6). Significance (*p < 0.05) between the groups (SNP *vs* control and 3% *vs* 21% O_2_) was established using a paired t-test.

Several studies have indicated that PE pathology is associated with placental hypoxia and oxidative stress^33,34^. To establish the mechanisms leading to the observed changes in SM-18:0 levels in PE apical membrane microdomains, we tested the effects of oxidative stress and low oxygen on the SM composition of placental lipid rafts. Human villous explants from 6–8 weeks gestation were exposed to sodium nitroprusside (SNP), a nitric oxide donor that induces oxidative stress, or cultured in a hypoxic environment of 3% O_2_. We are aware that 8% O_2_ would be the proper physiologic control for placental hypoxia experiments but to maximize the oxygen effect we exposed the villous explants to 21% O_2_. After 24 hours of exposure, explants were collected and DRMs (lipid rafts) were isolated. Sphingolipidomic analysis of the DRMs revealed that SNP treatment did not affect the content of any SM species while 3% O_2_ increased only the amount of SM-18:0 (Fig. 5A,B). Similarly to PE, we found that 3% O_2_, but not SNP-induced oxidative stress^15^, increased sENG content (Fig. 5C) as well as its interaction with SM-18:0 in the DRMs (Fig. 5D,E).

### Truncated endoglin is encapsulated in exosomes derived from apical (syncytial) lipid rafts

**Figure 6:**
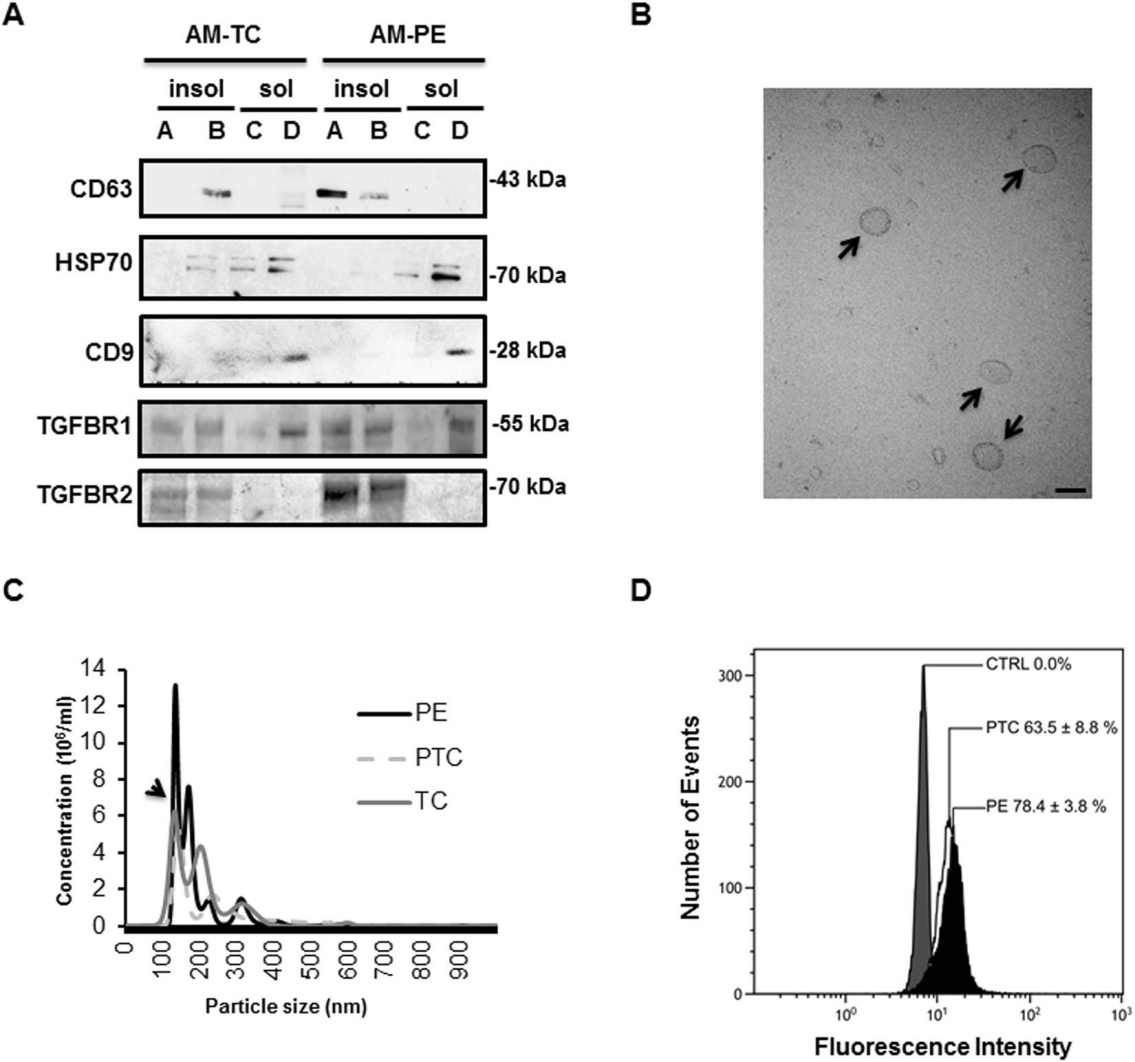
Placental circulating exosomes are derived from apical (syncytial) lipid rafts. (**A**) Immunoblotting for exosome markers (CD63, CD9, Hsp70), TGFBR1 and TGFBR2 of apical insoluble (**A**,**B**) and soluble (**C**,**D**) membrane fractions of PE and TC placentae. Experiment was repeated twice with similar results using different placentae. The images are cropped for clarity purposes. The full-length blots are presented in Supplementary Fig. 9. (**B**) Transmission electron microscopy (bar = 100 nm) and (**C**) particle size measurements of exosomes purified from maternal plasma. Experiment was repeated twice with similar results using different plasma samples. (**D**) Flow cytometry analysis of PLAP-expressing exosomes isolated from plasma of PE and normotensive PTC mothers. Data are shown as mean fluorescence shift compared to the negative control and values represent mean ± s.e.m. PE, n = 4 and PTC, n = 3 separate samples.

**Figure 7:**
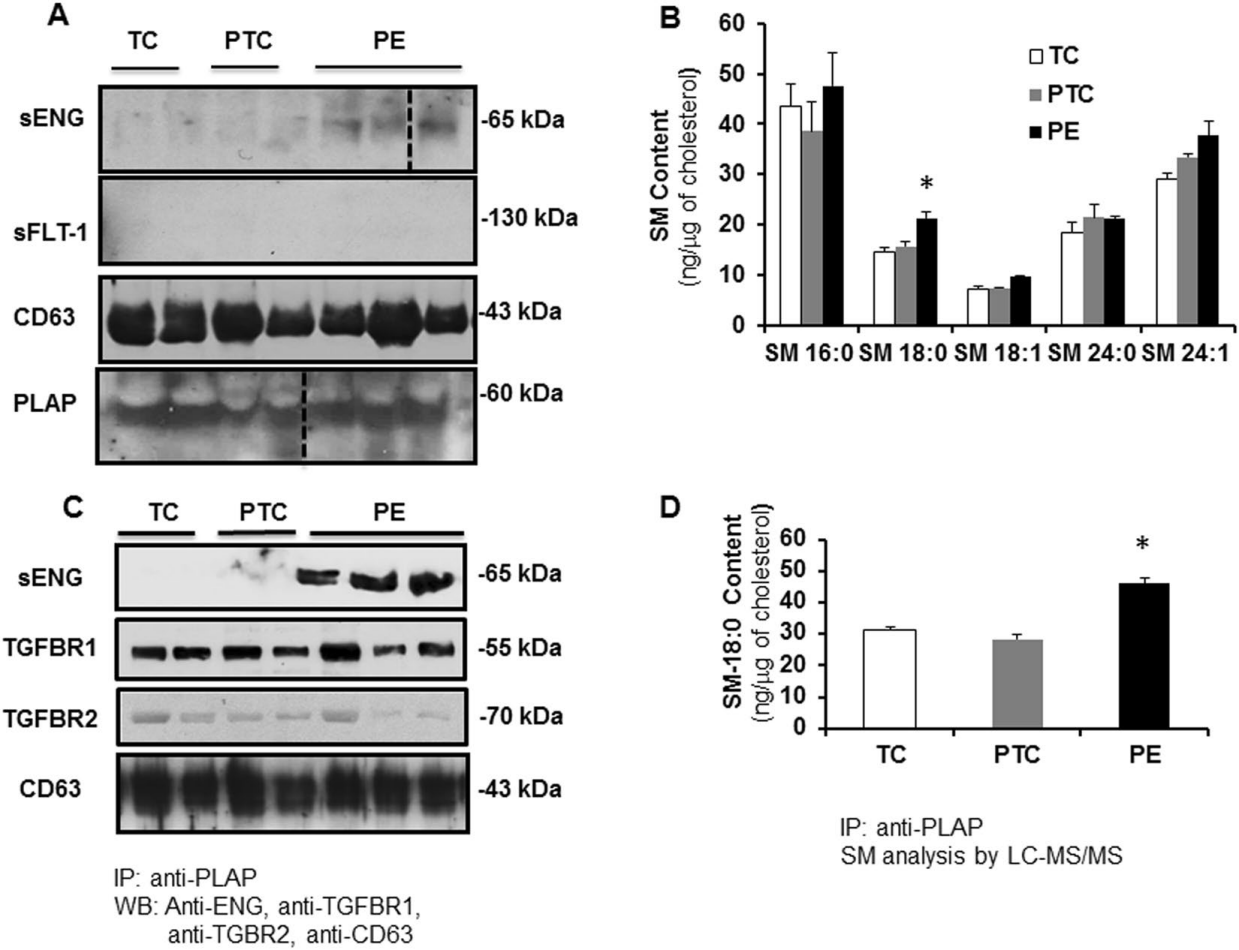
Truncated endoglin is encapsulated in circulating placental exosomes derived from apical (syncytial) lipid rafts. (**A**) Immunoblotting for sENG, sFLT1, CD63 and PLAP in exosomes isolated from TC (n = 2), PTC (n = 2) and PE (n = 3) maternal plasma. Experiment was repeated twice with similar results using different plasma samples. The images are cropped for clarity purposes. The full-length blots are presented in Supplementary Fig. 10. (**B**) SM species present in exosomes isolated from TC, PTC and PE maternal plasma (PE, n = 3; PTC, n = 3; TC, n = 3). Significance (*p < 0.05) was determined by one-way ANOVA for every single sphingomyelin species. (**C**) Immunoblotting for sENG, TGFBR1, TGFBR2 and CD63 of PLAP-precipitated exosomes from TC (n = 2), PTC (n = 2) and PE (n = 3) maternal plasma. Experiment was repeated twice with similar results using different plasma samples. The images are cropped for clarity purposes. The full-length blots are presented in Supplementary Fig. 10. (**D**) Quantification of SM-18:0 in PLAP-precipitated exosomes from TC, PTC and PE maternal plasma (PE, n = 4; PTC, n = 3 TC, n = 3). Data in (**B**,**D**) are presented as mean ± s.e.m. and significance (*p < 0.05) was determined by one-way ANOVA.

Throughout pregnancy syncytiotrophoblast secretes exosomes in the maternal circulation^35^. Lipid raft domains of the cell surface membrane are prone to outward bending or budding of exosomes^36^ as suggested by the high levels of cholesterol and sphingomyelin (major lipid raft constituents) in the exosomal lipid bilayer^37^. PLAP, the principal marker of placental derived exosomes, is also present in lipid rafts of apical (syncytial) membranes^24,36^, in agreement with these lipid rafts functioning as exosomal platforms. To examine whether the shortened ENG is secreted into the maternal circulation by the PE syncytium via lipid raft-derived exosomes, we first assessed the presence of exosome markers in DRMs of apical (syncytial) microvillous membranes. DRMs (insoluble fractions A and B) of apical (syncytial) membranes were indeed enriched in exosome markers such as CD63 (Fig. 6A)^36^. The DRMs stained weakly positive for CD81, another exosome marker (not shown). Surprisingly, the soluble fractions of apical (syncytial) membranes (the compartment in which sFLT1 resides (Fig. 4B)) were enriched in CD9 and HSP70, two other well-known exosome markers (Fig. 6A)^35^. We subsequently isolated exosomes from the maternal plasma of TC, PTC and PE pregnancies. Isolated exosomes were characterized by TEM (Fig. 6B), particle size (Fig. 6C), and immunoblotting for CD63 and PLAP (Fig. 7A). Our exosome preparation was enriched in vesicles with an average size of 100 nm. The vesicle size distribution did not differ between TC, PTC and PE maternal sera (Fig. 6C). Flow-cytometry using anti-PLAP revealed that a substantial portion (60–78%) of the total circulating exosomal vesicles were of placental origin (Fig. 6D). The percentage of PLAP-positive vesicles did not significantly differ between PE and PTC plasma. The enriched exosomal vesicle fractions were then subjected to SDS-PAGE followed by immunoblotting for sENG and sFLT1. The truncated ENG was present in the exosomes isolated from maternal plasma from PE pregnancies, but not in circulating exosomes from PTC and TC pregnancies (Fig. 7A). Notably, none of the exosomes contained sFLT1 (Fig. 7A). Lipidomic analysis revealed that the exosomes contained abundant sphingomyelins and cholesterol. Importantly, exosomes circulating in maternal sera of PE patients were significantly enriched in SM-18:0 compared to exosomes from TC and PTC maternal blood (Fig. 7B). CD63-enriched exosomes isolated from circulating PE exosomes by anti-CD63 immunoprecipitation also contained significantly more SM-18:0 than CD63-enriched exosomes from TC plasma (Supplementary Fig. 5A). Similarly, placental-derived exosomes obtained by anti-PLAP immunoprecipitation from total circulating PE exosomes were enriched in sENG as well as SM-18:0 when compared to PLAP-precipitated exosomes from TC and PTC plasma (Fig. 7C,D). We also probed DRMs of apical (syncytial) microvillous membranes and circulating placental-derived exosomes for other elements of the TGFB receptor complex. Independent of preeclampsia, apical insoluble DRMs and placental-derived (PLAP-immunoprecipitated) exosomes contained the type 1 receptor TGFBR1 (ALK5) (Figs 6A and 7C). The type 2 receptor TGFBR2 was present in the DRMs (Fig. 6A) and to a lesser extent also in the exosomes (Fig. 7C). The type 1 receptor ALK1 was absent from both compartments (not shown).

## Discussion

Herein we demonstrate that the molecular distribution of sphingomyelins is altered in DRMs of apical (syncytial) microvillous membranes of preeclamptic *vs* preterm and term control placentae; in particular, SM-18:0 content is markedly increased. Using human villous explants we found that low oxygen, but not SNP-induced oxidative stress, is responsible for the increased SM-18:0 content. Directly (PLO assays) and indirectly (immunoprecipitation followed by LC-MS/MS) we show a unique interaction between ENG, MMP14 and SM-18:0. ENG partially clusters with MMP14 in SM-18:0 enriched membranes of the Golgi network and is then transported to the apical microvillous membranes of PE placentae where ENG gets cleaved, most likely by MMP14^7^, into a shorter form of ENG. Of clinical relevance, we show that this shortened ENG is present in SM-18:0 enriched placental exosomes isolated from maternal blood of PE patients. In contrast, soluble FLT1 is present in the non-lipid raft region of apical PE membranes and absent in circulating placental exosomes of PE mothers. These data are compatible with sENG and sFLT1 being released by the PE syncytium *via* different mechanisms and circulating in separate compartments in the maternal blood. Furthermore, the data reveal that circulating endoglin is in fact not soluble but present in exosomes.

In the present study we found that sENG was the predominant ENG protein present in SM-18:0 enriched lipid rafts of apical (syncytial) membranes of PE placentae. Also MMP14 localized to these apical SM18:0 enriched lipid rafts. Previous studies have shown that MMP14 localizes to the syncytiotrophoblast^7^ and is present in lipid rafts of human cancer and endothelial cells^38,39^. Based on the co-localization of MMP14 and sENG we propose that full-length ENG is cleaved by MMP14 within these lipid rafts at the syncytial trophoblast cell surface. This concept is corroborated by our observation that the Golgi compartment of PE placentae mainly contained the full-length ENG that co-localized with MMP14 to SM-18:0 enriched microdomains within PE Golgi membranes. However, the absence of the truncated ENG in the Golgi complex suggest that MMP14 is not active in this compartment. Importantly, we found that in normotensive control placentae, full-length ENG is solely present in the non-raft areas of the plasma membrane, which are devoid of MMP14. This observation and the exclusive presence of full-length ENG in the non-raft compartment of the apical PE plasma membrane, lends further support for MMP14 cleavage of ENG only in the lipid rafts.

The Golgi apparatus is the major cellular site of SM synthesis^40^. SM synthases (SMS1 and 2), which are both present in the *trans*-Golgi, catalyze the final step in the SM synthetic pathway, namely the transfer of the phosphocholine headgroup from phosphatidylcholine onto ceramide thus producing sphingomyelin and diacylglycerol^41^. Since SM synthases appear promiscuous towards their ceramide substrate, an increase in ceramide availability is likely responsible for the increase in SM-16:0 and SM-18:0 in PE placentae. Ceramides are produced in the endoplasmic reticulum and then transported to the Golgi apparatus^42^. Recently, we demonstrated increased serine-palmitoyl transferase activity, the rate limiting enzyme in *de novo* ceramide synthesis, in PE placentae^15^. This finding in conjunction with the increase in C16:0 and C18:0 ceramides in the Golgi (Supplementary Fig. 4F) supports the idea of increased ceramide availability for SM synthases to produce SM-16:0 and SM-18:0 in PE placentae. Whether these changes are due to the hypoxic milieu is unknown. It has been reported that dihydroceramide desaturase activity is decreased by hypoxia leading to the accumulation of dihydroceramide^43^. However, the effect of hypoxia on placental ceramide synthase 1 that synthesizes C18:0 ceramide (the precursor for SM-18:0) is not known and we therefore can only speculate that its activity is increased.

Lipid rafts in the Golgi complex are involved in sorting of lipids and proteins for transport from the *trans*-Golgi network (TGN) to cell surface membranes^44^. TGN lipid domains enriched in sphingolipids and cholesterol are preferentially sorted to the apical surface membrane^45^ together with proteins clustering in these lipid raft platforms due to their affinity for sphingolipids and cholesterol^19^. Thus, it is plausible that increased SM-18:0 production in the *trans*-Golgi complex of PE placentae promotes clustering of ENG and MMP14 in SM18:0 enriched lipid rafts of the Golgi leading to subsequent sorting to the lipid rafts of the apical cell surface membrane. However, in PE placentae, full-length ENG was also targeted to the non-raft compartments of the apical membrane, which were not enriched in SM18:0 (data not shown). ENG did not associate with any particular SM in this compartment (Supplementary Fig. 2D), suggesting that not all ENG in the trans-Golgi was sequestered in the SM-18:0 enriched lipid compartment. *In silico* analysis did not reveal any known sphingolipid-binding motif in ENG and MMP14^46^.

MMP14 (MT1-MMP) belongs to a large family of matrix metalloproteinases^47^. It degrades extracellular matrix components^48^, activates or inactivates various cytokines and chemokines^49,50^, and cleaves cell surface receptors^51,52^, including ENG^7^. Activation of MMP14 seems to be accomplished intracellularly by furin, a serine proteinase of the *trans*-Golgi network, which recognizes the unique amino acid sequence (RRKR) located between the pro and catalytic domain of MMP14^53^. Once activated, MMP14 is immediately transported to the plasma membrane^54^. In the current study, we did not observe any active MMP14 in the Golgi complex of preeclamptic placentae, which is likely due to the rapid transfer of activated MMP14 to the surface membrane. Here, active MMP14 then cleaves full-length ENG to sENG, the only ENG detectable in the SM-18:0 enriched lipid rafts of the apical syncytial microvillous membranes.

In the present study we co-localized SM-18:0 and ENG to syncytial knots of the PE placenta. Syncytial knots are derived from aged or damaged regions of the syncytiotrophoblast that are extruded into the maternal blood stream^55^. The placental ST secretes a wide range of micro- and nano-sized vesicles into the maternal circulation during normal pregnancy^56^. High amounts of syncytiotrophoblast-derived extracellular vesicles have been found in plasma from women with early-onset PE^57^. It has been suggested that in normal pregnancy the placenta constitutively releases small exosomes while in PE oxidative and inflammatory stress stimulates the ST to shed larger microvesicles^58^. In the present study, however, we did not observe any size differences between the physiological and pathological derived exosomes. The molecular composition and biological cargo of these extracellular vesicles is determined by their cellular origin. It has been reported that exosomal protein content is different in women with preeclampsia. For example, syncytin-2 is reduced in serum-derived exosomes from women with PE when compared to exosomes from normal pregnant women^57^. Our lipodomic analysis indicated a significant increase of SM-18:0 in plasma of preeclamptic women compared to normotensive controls. In addition, we found that the shortened ENG was present in SM18:0-enriched placental exosomes in the circulation of PE women. Notably, SM-18:0 enriched lipid rafts of apical membranes from PE placentae contained sENG and CD63. The latter is a well-known placental exosome marker^35^. Thus, we postulate that the SM-18:0-enriched apical lipid rafts of the syncytiotrophoblast are the platforms for assembling and secreting into the circulation SM-18:0 enriched exosomes that contain sENG. Although it has been suggested that sFLT1 is present in ST-derived microvesicles^59,60^ our placental circulating exosomes were devoid of sFLT1. This opposite finding is likely due to differences in isolation procedure of exosomes and argues for a standardized methodology for comparing results.

Our present data show that besides sENG placental-derived exosomes isolated from the preeclamptic maternal blood contained TGFBR1 (ALK5) and TGFBR2, but not the type 1 receptor ALK1. The absence of ALK1 in the circulating placental-derived exosomes confirms that these microvesicles originated from syncytiotrophoblast and not from placental endothelial cells, which express ALK1^14^. We hypothesize that sENG in the exosomes, in association with TGFBR1 and TGFBR2, bind TGFB and block its effects. A truly soluble form of ENG cannot bind TGFB under physiological conditions^61^. Therefore, we propose that the circulating form of endoglin associated with preeclampsia^3^ is not a soluble protein. It is a truncated extracellular domain of ENG (likely cleaved by MMP14^7^), secreted from the syncytiotrophoblast *via* exosomes together with both TGFB receptors.

The discovery of increased circulating levels of the truncated ENG and sFLT1 in preeclampsia has led to investigations into their utility as potential early gestation biomarkers^62^. However, their value as early predictors has been difficult to assess - possibly because of the presence of a variety of circulating proteins of both maternal and placental sources in the plasma of pregnant women. Considering that exosomes isolated from the circulation of normotensive women do not contain sENG, the detection of shortened ENG together with SM-18:0 in exosomes of placental origin during pregnancy could potentially be a more accurate and specific biomarker set for early diagnosis of preeclampsia.

**Figure 8:**
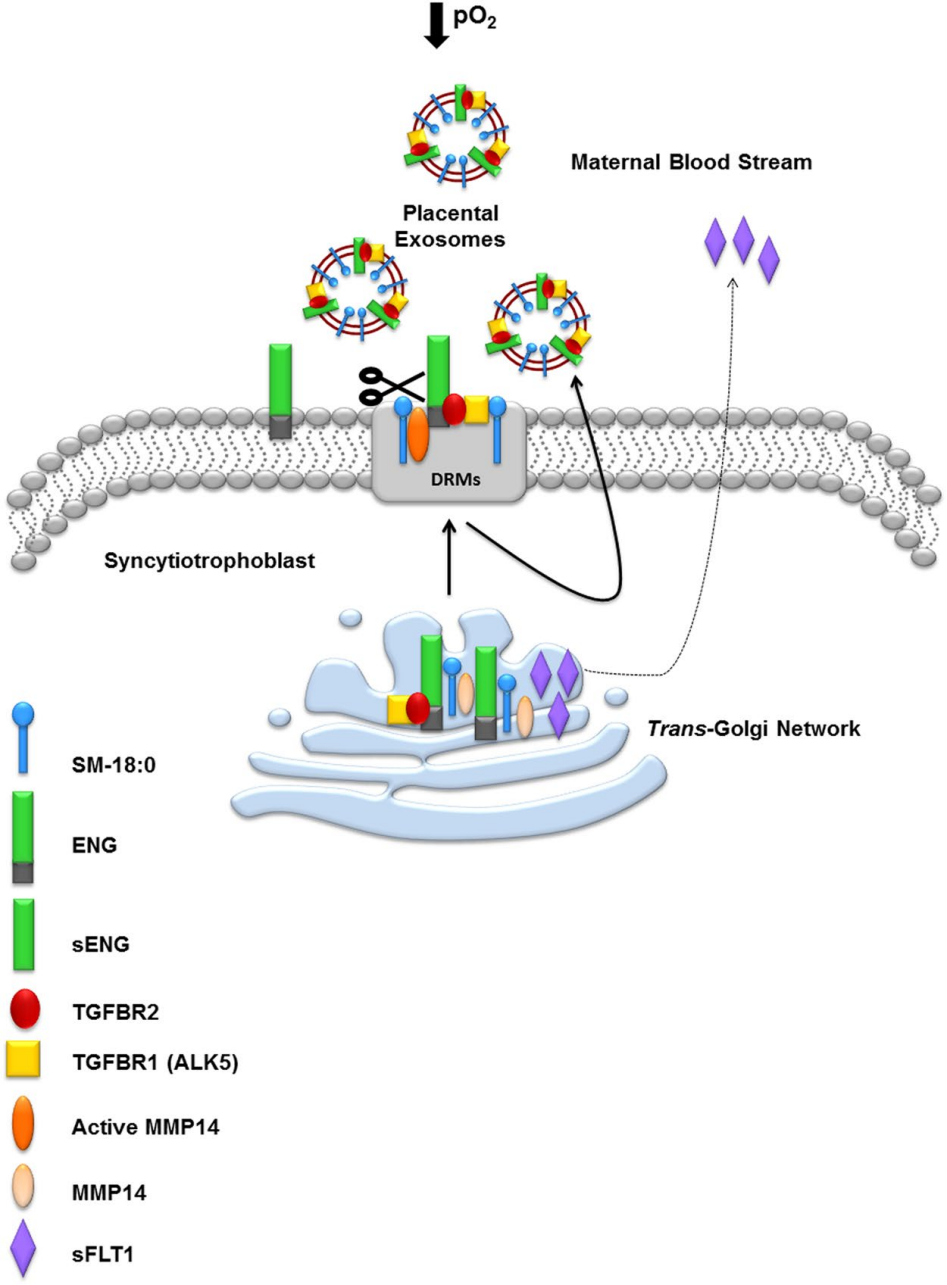
Schematic representation of the shedding of truncated ENG (sENG) and soluble FLT1 in PE placenta. The hypoxic environment of the preeclamptic placentae increases SM-18:0 and its association with ENG, MMP14, TGFBR1 and TGFBR2 in the lipid rafts of the trans-Golgi network. The SM-18:0 lipid domains of the TGN are then targeted to the cell surface membrane of syncytiotrophoblast where active MMP14 cleaves ENG into sENG. The apical SM18:0-enriched microdomains containing sENG and the TGFB receptors are secreted as exosomes into the maternal circulation. sFLT1 is synthesized as a splice variant of the vascular endothelial growth factor receptor FLT1 that lacks the transmembrane domain. It does not locate to the SM-18:0 lipid raft domains in the TGN and is not present in circulating placental exosomes, suggesting it is secreted either as a soluble protein or in a separate microvesicle in the blood torrent.

In conclusion, the hypoxic environment typical of preeclamptic placentae stimulates the formation of SM-18:0, leading to SM-18:0 enriched lipid rafts in the *trans*-Golgi network. ENG and MMP14, but not sFLT1, have an affinity for sphingolipids and cluster in these SM-18:0 enriched lipid rafts of the TGN. The SM-18:0 lipid domains of the TGN are then targeted to the cell surface membrane of syncytiotrophoblasts where MMP14 becomes active and cleaves ENG into sENG. These l SM18:0-enriched microdomains containing sENG, TGFBR1 (ALK5) and TGFBR2 are then secreted as exosomes into the maternal circulation where potentially they could scavenge circulating TGFB (see model depicted in Fig. 8).

## Methods

### Placental tissue and plasma collection

Informed consent was obtained from each individual. Tissue and serum collection was carried out in agreement with collaborating institutions’ ethics guidelines (Ethics guidelines of the University of Toronto’s Faculty of Medicine, Mount Sinai Hospital and O.I.R.M Sant’ Anna Hospital, University of Turin, Italy). The experimental protocol has been approved by the Mount Sinai Hospital Research Ethics Board (REB number: 11-0287-E). First trimester human placental tissues (6–8 weeks of gestation, n = 20) were obtained immediately following the elective termination of pregnancies by dilation and curettage, or suction evacuation, and were used for villous explant cultures. Gestational age was determined by the date of the last menstrual period and first trimester ultrasound measurement of crown-rump length. The study group including women experiencing preeclampsia (PE, n = 87) was selected on the basis of ACOG clinical and pathological criteria^63^. Calcified, necrotic and visually ischemic areas of the placental tissue were omitted from specimen. Placental tissues obtained from age-matched preterm (PTC, n = 75) and term (TC, n = 28) deliveries from healthy pregnancies with normally grown fetuses that did not have signs of preeclampsia or other placental dysfunctions were included as controls. Placental samples were obtained and pooled from 4 placental quadrants representative of the whole placenta and did not contain umbilical cord insertion sites, areas of obvious thrombosis or infarcts as well as other abnormalities. After harvesting, tissue samples were pooled and immediately snap-frozen in cryovials in liquid nitrogen for further analysis. Plasma samples were obtained prior to delivery from women exhibiting clinical signs of early onset preeclampsia (PE, n = 16) and from normotensive control patients (PTC, n = 13; TC, n = 13). Plasma samples were frozen and stored at −80 °C. Patients’ information and clinical parameters are outlined in Table 1.

**Table 1:** Clinical parameters of the study population.

	PTC	PE	TC
	(n = 75)	(n = 87)	(n = 28)
Gestational Age at Delivery	30.6 ± 2.6	29.8 ± 2.0	38.5 ± 0.6
Blood Pressure (mm Hg)	S:117.1 ± 3.9/	S:168.5 ± 12.4	S:111.5 ± 4.8
	D:68.8 ± 4.9	D:105.5 ± ±9.6	D:68.2 ± 4.9
Proteinuria (g/day)	Absent	3.16 ± 1.24	Absent
Fetal Weight (g)	1623.0 ± 329.0	1284.2 ± 447.4	3679.6 ± 222.6
		AGA: 69.1%	AGA: 100%
		IUGR: 30.9%	
Fetal Sex	M: 58.5%	M: 63.3%	M: 76.0% F:24.0%
	F: 41.5%	F: 36.7%	
Mode of delivery (%)	CS: 44.6%	CS: 82.5%	CS: 84.0%
	VD: 55.4%	VD: 17.5%	VD:16.0%

Data are presented as mean ± SD. PTC: Preterm Controls, TC: Term Controls, PE: Preeclampsia, S: Systolic; D: Diastolic, M: Male; F: Female, CS: Caesarian Section, VD: Vaginal Delivery.

### First trimester villous explant culture

Chorionic villous explant culture was performed as previously described^64^ (see supplementary methods for detailed description).

### Protein Lipid Overlay Assay

A protein lipid overlay assay assay, adapted from Dowler *et al*.^65,66^ was used to identify lipids that interact with ENG (see supplementary methods for detailed description).

### Isolation of Placental Apical Membranes

Apical microvillous membranes were prepared from fresh human placentae according to the method of Jimenez *et al*.^27^. Placentae were obtained and processed immediately after delivery. Villous tissue (0.5 g) was chopped into small pieces, washed with saline and filtered through gauze to remove residual blood. The tissue was homogenized using a polytron PT10/35 (setting 3, 3 times for 30 s) in 3 volumes of ice-cold buffer A (250 mM sucrose, 0.7 × 10^−3^ mM pepstatin, 1.1 × 10^−3^ mM leupeptin, 0.8 × 10^−3^ mM antipain, 80 × 10^−6^ mM aprotinin and 10 mM Tris-HEPES, pH 7.4). The homogenate was centrifuged at 5,860 g for 15 minutes and supernatant was collected. The pellet was resuspended in buffer A, homogenized and centrifuged at 5,860 g. The supernatants were combined and centrifuged at 10,000 g for 15 minutes. The pellet was discarded and the supernatant was centrifuged at 124,000 g for 30 minutes. The resulting pellet was resuspended in 250 μl buffer A using a glass-teflon homogenizer. Magnesium chloride was added till an end concentration of 12 mM and the homogenate was stirred on ice for 20 minutes before centrifugation at 2,500 g for 10 minutes. The Mg2+ treatment separates the apical microvillous membranes (supernatant) from the basal membranes (pellet). The supernatant, containing the apical microvillous membranes, was centrifuged at 12,100 g for 70 minutes and the ensuing pellet, was resuspended in buffer B (300 mM sucrose, 20 mM Tris-maleate, pH 7.4). The pellet enriched in basal membranes was resuspended in 250 μl buffer A, stirred on ice for 60 minutes and centrifuged at 4,340 g for 10 minutes. The supernatant was centrifuged at 98,500 g for 45 minutes in a Beckman TL-100 ultracentrifuge and the resulting pellet containing the microsomal basal membranes, was resuspended in buffer A.

### Isolation of Detergent-Resistant Membranes

Total TC, PTC and PE placentae or their isolated apical membranes were homogenized in TNE buffer (25 mM Tris-HCl, pH 7.4, 150 mM NaCl, 2 mM EDTA) containing 2% (v/v) Triton X-100 using a Polytron tissue grinder (three 10-sec bursts with 20-sec intervals). The homogenates (0.58 ml) were then rotated for 30 minutes at 4 °C, adjusted to 40% (w/w) sucrose with 0.82 ml of 62% (w/w) sucrose, loaded into the bottom of MLS-50 centrifuge tubes (Beckman Coulter Canada, Mississauga, ON) and overlaid sequentially with 35%, 30%, 25%, 20%, 15%, and 10% (w/w) sucrose solutions (0.6 ml each). Samples were then centrifuged at 160,000 *g* for 18 hours at 4 °C in a TL-100 Beckman centrifuge. After centrifugation, eight 600 μl fractions were collected from top to bottom of the gradient and characterized as decribed in the supplementary sections.

### Isolation of Golgi Complex

The isolation of the Golgi apparatus from placental tissues was performed as described by Taylor *et al*.^67^ (see supplementary methods for detailed description).

### Exosome Isolation

To isolate exosomes, 250 μl of plasma was mixed with 67 μl ExoQuick (System Biosciences, Mountain View, CA) and incubated for one hour at 4 °C. Precipitated vesicles were then collected by centrifugation (30 minutes at 1500 *g*), suspended in 2.5 M sucrose and subjected to a discontinuous 0.25–2.5 M sucrose gradient centrifugation (110,000 *g* for 20 hours in a Beckman TL-100 ultracentrifuge). Fractions (10 in total, 300 μl each) were collected and exosomes were characterized by Nanosight size measurements, TEM, FACS and immunoblotting for exosome marker proteins (see supplementary methods for detailed description).

### Immunoprecipitation Analysis

#### Placental tissue lysates

Whole tissue lysates were pre-cleared using Protein G-sepharose. Lysates were then incubated overnight with antibodies against either ENG (H-300 antibody) or FTL-1. Antibodies were used in the dilutions stated below. Protein G-Sepharose was used to precipitate the immune complexes. The complexes were dissociated by heating in sample buffer (10% glycerol, 2% SDS, 5% β-mercaptoethanol, 0.0025% bromophenol blue, 0.06 M Tris, pH 8.0) and subjected to both Western blotting for ENG (P4A4 antibody) and FLT-1 as well as lipidomic analysis. *DRMs and Exosomes*: Isolated DRMs and exosomes were suspended in 0.3 M sucrose, 1.5 mM MgCl, Tris, pH 7.4, pre-cleared using Protein G-Sepharose and incubated overnight with either anti-ENG (H300), anti-MMP14, anti-CD63 or anti-PLAP. Antibodies were used in the dilutions stated below. Protein G-Sepharose was added and the mixture incubated for 4 hours at 4 °C. Beads were pelleted and washed 6 times in excess incubation buffer. The complexes were then subjected to Western blotting for ENG (P4A4 monoclonal antibody), CD63 and MMP14 as well as to lipidomic analysis. *Golgi complex*: Isolated Golgi complex was suspended in 0.3 M sucrose, 1.5 mM MgCl, Tris, pH 7.4 containing 0.2% Triton and incubated overnight with anti-ENG (H300) antibody or anti-MMP14 antibody. Antibodies were used in the dilutions stated below. Protein G-Sepharose was added and the mixture incubated for 4 hours at 4 °C. Beads were pelleted and washed 6 times in excess incubation buffer. The complexes were then subjected to lipidomic analysis.

### Endoglin Measurement in Plasma

ENG in plasma from PE and PTC mothers was measured using a Human Endoglin/CD105 Quantikine ELISA kit according to the recommendations of the manufacturer (R&D Systems, Inc. Minneapolis, MN, USA).

### MALDI-Mass Spectral Imaging

Placental tissues from PE and PTC pregnancies were processed for MALDI-mass spectrometry imaging (MALDI-MSI) as described previously^15^. See Supplementary Materials for details. After MALDI imaging, the matrix was removed and the tissue fixed with 4% paraformaldehyde prior to H&E staining or standard immunohistochemistry for ENG as previously described^64^.

### Lipid Mass Spectral Analysis

Placental tissues, sucrose gradient fractions, sera and immunoprecipitates from PE, PTC and TC pregnancies were processed for lipid extraction. Following extraction, sphingolipids and cholesterol were quantified using high performance liquid chromatography and tandem mass spectrometry (LC-MS/MS) at the Analytical Facility for Bioactive Molecules of the Hospital for Sick Children, Toronto, ON. See Supplementary Materials for details.

### Statistical analysis

Statistical analysis was performed using GraphPad Prism 6 (GraphPad Software, Inc) software using Student’s t-test or one-way ANOVA followed by posthoc Dunnet test where applicable. Data are expressed as the mean ± SEM and a value of P < 0.05 was considered statistically significant (*P < 0.05) Results are expressed as mean ± s.e.m.

## Electronic supplementary material

Supplemental Methods and Data
