# Supplementary material for: A Single Sphingomyelin Species Promotes Exosomal Release of Endoglin into the Maternal Circulation in Preeclampsia
Source: Sci Rep. 2017 Sep 22;7:12172. doi: 10.1038/s41598-017-12491-4 (PMC5610344; doi:10.1038/s41598-017-12491-4)
Supplement: Supplementary file 1 — Supplemental Methods and Data [file 41598_2017_12491_MOESM1_ESM.pdf]

A Single Sphingomyelin Species Promotes Exosomal Release of Endoglin into the Maternal Circulation in Preeclampsia

Leonardo Ermini, Johnathan Ausman, Megan Melland-Smith, Behzad Yeganeh, Alessandro Rolfo, Michael Litvack, Tullia Todros, Michelle Letarte, Martin Post, Isabella Caniggia

Supplementary Materials

*

Detailed Methods

First trimester villous explant culture. Chorionic villous explant culture was performed as described previously [1]. Briefly, human placental tissue was aseptically dissected to remove decidual components and fetal membranes. Small fragments of placental villi were dissected and cultured in serum-free DMEM/F12 (GIBCO-BRL, Grand Island, NY) supplemented with 10,000 units/ml of penicillin/streptomycin.

Oxygen experiments: Villous explants were cultured in either ambient (21% O_2) air or in an atmosphere of 3% O_2/92% N_2/5% CO_2. After 24 hours, explants were collected and detergent-resistant membranes were isolated.

SNP treatment: Villous explants maintained in ambient air were treated with and without sodium nitroprusside (SNP: 2.5 and 5.0mM; Sigma-Aldrich) for 24 hours, followed by isolation of detergent-resistant membranes.

Protein Lipid Overlay Assay (PLO). A PLO assay, adapted from Dowler *et al.* [2] was used to identify lipids that interact with ENG. Briefly, serial dilutions of different lipid species (2.5-100 pmol) were spotted onto a nitrocellulose membrane. These membranes were then incubated overnight in PLO buffer (50 mM Tris–HCl, pH 7.5, 150 mM NaCl, 3% BSA and 0.06% Tween) that contained either commercial GST-conjugated full-length ENG or His-conjugated extracellular-domain encompassing ENG (Met1-Gly586). The membranes were washed with PLO buffer and ENG bound to the membrane by virtue of its interaction with lipid(s) was then detected by immunoblotting with an antibody recognizing the tagged epitope (GST or His) or with the polyclonal H300 antibody to ENG.

Characterization of Sucrose Gradient Fractions. The flotation gradient fractions were characterized using specific protein and/or lipid markers. Placental alkaline phosphatase (PLAP) activity was used as a marker for apical (syncytial) DRMs (also called lipid rafts) while caveolin-1 and flottilin-2 were used as markers of caveolae and lipid rafts, respectively. Lipid raft markers such as cholesterol and GM1 (monosialotetra hexosylganglioside) were also measured. Human transferrin receptor (TFRC) and calnexin were used as non-lipid raft markers.

PLAP activity: Placental alkaline phosphatase (PLAP) activity was assayed by incubating membrane fractions with a buffer containing 67 mM Tris-HCl, pH 8, 1 mM MgCl2, and 1 mM p-nitrophenylphosphate for 10 minutes at 37 °C. After stopping the reaction with NaOH, formation of para-nitrophenol was measured at 405 nm.

GM1 detection: To measure the levels of GM1, an aliquot (3 μl) of each gradient fraction was spotted on nitrocellulose membrane, dried for 1 hour and then blocked with 3% (w/v) BSA in PBS (128 mM NaCl, 2 mM KCl, 8 mM Na2HPO4, 2 mM K2HPO4, pH 7.2) for 2 hours at room temperature. The membrane was incubated overnight with biotin-conjugated cholera toxin B subunit followed by incubation with horse peroxidase (HRP)-conjugated streptavidin. Membrane blots were visualized using enhanced chemiluminescence (PerkinElmer Inc., Waltham, MA, USA) and imaged on X-ray film (GE Healthcare).

Cholesterol measurements: Cholesterol distribution between the fractions was determined by thin layer chromatography (TLC). Lipids were extracted from equivalent aliquots of all fractions and separated on Silica Gel 60 F-254 plates (Millipore Merck, Etobicoke, ON) with chloroform:methanol (98:2, v/v) as solvent phase. Cholesterol was visualized by spraying plates with 20% (v/v) H2SO4 and exposure to 150°C. Cholesterol in the soluble and insoluble fractions was quantified by LC-MS/MS.

Cholesterol Depletion: Placental tissues were homogenized in TNE buffer containing 1% (v/v) saponin and incubated for 60 min at room temperature with rotation. The DRMs were then isolated as described above.

Isolation of Golgi Complex. The isolation of the Golgi apparatus from placental tissues was performed as described by Taylor et al. [3]. Placentae obtained from preeclamptic and normotensive control pregnancies were finely minced, and wet weight was determined. The minced tissue was resuspended in 600 mg/ml of 0.5 M sucrose containing 100 mM KH2PO4/K2HPO4, pH 6.8, 5 mM MgCl2, and 4 mg of proteolytic inhibitors (chymostatin, leupeptin, antipain, and pepstatin). All other sucrose solutions mentioned below contained the same buffer and proteolytic inhibitors. The tissues were homogenized using a Polytron PT10/35. The homogenate was centrifuged at low speed (1,500g for 10 minutes) to pellet unbroken cells, cell debris, and nuclei. The resulting post-nuclear supernatant (PNS) was loaded in the middle of a sucrose step gradient in an MLS-50 tube (Beckman Coulter Canada, Mississauga, ON): layers 1 and 2 of 1.3 M and 0.86 M sucrose, respectively, were overlaid with the PNS, followed by a layer of 0.25 M sucrose. The gradient was centrifuged at 100,000g for 60 minutes in a TL-100 ultracentrifuge (Beckman Instruments, Palo Alto, CA). The following fractions were collected from the top of the gradient by using a wide-bore transfer pipet: SI, 0.25– 0.5 M interface; A, 0.5 M layer; SII, 0.5– 0.86 M interface (Golgi containing fraction); B, 0.86 M layer; SIII, 0.86 –1.3 M interface; C, 1.3 M layer; and pellet. The purity of Golgi fraction was checked by Western blotting analysis using anti-Golgin 97 (see Supplemental Fig. 3A for PE Golgi characterization). The level of contamination of the Golgi fraction was evaluated using an anti-pan-cadherin antibody; marker of plasma membrane.

Characterization of Exosomes. Nanoparticle Size measurements: The size of the isolated exosomes was measured using a NanoSight NS 300 particle analyzer (Malvern Instruments Ltd, Malvern, UK). Briefly, exosomes were sedimented by ultracentrifugation (200,000 g for 20 h in Beckman TL-100), suspended in 100 µl of PBS, diluted 1:10,000 and characterized with the NS300 equipped with Nanoparticle Tracking Analysis (NTA) software.

Tra*nsmission electron microscopy (TEM):* For TEM analysis, exosomes were sedimented by ultracentrifugation (200,000 g for 20 h in Beckman TL-100) from the appropriate sucrose gradient fractions and fixed in 3% (w/v) glutaraldehyde and 2% paraformaldehyde in cacodylate buffer, pH 7.3. The fixed exosomes were then applied to a continuous carbon grid and negatively stained with 2% uranyl acetate. The samples were examined with a Philips CM10 electron microscope.

PLAP FACS analysis: To determine the percentage of PLAP-expressing exosomes for a given condition we used Exo-Flow kits for FACS analysis (System Biosciences, Mountain View, CA). Briefly, the exosomes were precipitated from human blood plasma samples using ExoQuick as described above. For each sample, 100 µl of isolated exosomes were added to magnetic beads coupled with anti-PLAP antibody and incubated on a rotating rack at 4°C overnight. The beads were then incubated with Exo-FITC exosome stain on ice for 2 hours, washed and analyzed by flow cytometry on a Beckman Coulter Gallios 10/3 flow cytometer. Data was analyzed using the Beckman Coulter Kaluza software package. All flow cytometry acquisitions consisted of 10,000 events each representing 1 bead per event. Fluorescence intensity shift was compared to a bead preparation that included FITC label but did not include exosomes (negative control).

Mass Spectral Analysis of Spingolipids

Lipid extraction procedure: Placental tissues were collected and kept at -80°C until lipid extraction. Twenty five mg of frozen tissue was lyophilized, transferred to siliconized glass tubes and homogenized in 2 mL of (1:1) methanol/water. Samples were spiked with a mixture of internal standards (cholesterol-d7; 5 ng) (17:0 ceramide (d18:1/17:0), 17:0 sphingomyelin (d18:1/17:0); 10 ng of each (Avant Polar Lipids, Alabaster, AL)). After addition of 2 mL of chloroform, samples were vortexed for 1 minute, kept on ice for 10 minutes, and then centrifuged at 1,000*g* for 5 minutes. The chloroform layer was collected and dried under a stream of nitrogen. Samples were then reconstituted in 100 µl ethanol acidified with 2 µL of formic acid and transferred to siliconized minivials for analysis by LC-MS/MS. Serum samples (25 µl) were spiked with a mixture of aforementioned internal standards and then extracted and prepared for LC-MS/MS as described above.

Mass spectrometry: Liquid chromatography coupled tandem mass spectrometry (LC-MS/MS) for sphingolipids was performed on an Agilent 1200 Series binary pump (Agilent Technologies Inc., Santa Clara, CA) coupled to an API4000 triple-quadrupole mass spectrometer (Sciex, Concord, ON). Prior to analysis, Multiple Reaction Monitoring (MRM) mass transition parameters were optimized by infusion of pure standards (5 µl/minute of 1ug/mL). Reverse phase high performance liquid chromatography (HPLC) was performed using a Kinetex C18 column (2.6 µm, 100x2.1 mm; Phenomenex, Torrance, CA). Sample injection volume was 1-5 µL. The mobile phase consisted of (A) water/acetonitrile/methanol (2/1/1, v/v/v) and (B) tetrahydrofuran/acetonitrile/ methanol (2/1/1, v/v/v) with both components containing 0.05% formic acid. At a flow of 400 µL/minute the HPLC gradient was as follows: initial conditions of 60:40 (A:B) were held for 4.5 minutes prior to injection, held for another 2 minutes post

injection and ramped to 15:85 (A:B) over 13 minutes. Conditions were held for 15 minutes and returned to initial conditions for 17 minutes. MS analysis was performed in positive electrospray Ionization mode. The source temperature was maintained at 400°C with the ion spray voltage set at 5,000 V and nitrogen used as the Collision Induced Dissociation gas. MRM Mass Transitions and Chromatographic Retention Times of sphingolipids have been published previously.[4] For quantitative analysis a separate standard curve was generated for each analyte measured using MRM area ratios (Analyte Peak Area/IS Peak Area). Results were then calculated by plotting the sample area ratios against their respective analyte specific standard curve [5,6].

For cholesterol quantification, LC-MS/MS was performed on an Agilent 1290 HPLC System (Agilent Technologies: Santa Clara, California, USA) coupled to a Sciex 5500 QTRAP Mass Spectrometer (Sciex, Concord, ON). Chromatography ran at a flow rate of 500 µL/minute on a Phenomenex Kinetex XB-C18 column (2.6 µm, 50x3.0 mm (Phenomenex, Torrance, CA)) isocratically at 100% methanol + 0.1 % formic acid over 4 minutes. The mass spectrometer was operated in positive APCI mode with the following settings: source temperature = 600°C, curtain gas = medium, DP = 40, EP = 10, CE = 30, CXP = 11. Precursor to product ion mass transitions was established using standard infusions. Data was acquired using the following MRM transitions; cholesterol ($-H_2O+H$) 369.4->135.1, 161.1 m/z, cholesterol-d7 ($-H_2O+H$) 376.4->135.1, 161.1 m/z; the 135.1 product ions were used for quantitation. Data integration and quantitation was performed using Sciex Analyst 1.6 software.

MALDI-Mass Spectral Imaging of Sphingolipids

Reagents: Polyvinyl alcohol (PVA) 6-98, 2,5-dihydroxybenzoic acid (DHB), polypropylene glycol with average molecular weight 2000 g/mol (PPG 2000) and sodium azide were purchased

from Sigma Aldrich (St. Louis, MO). Optimal cutting temperature (OCT) compound was obtained from Leica Microsystems (Heidelberg GmbH, Mannheim, Germany). Organic solvents were purchased from Caledon Laboratory Chemicals (Georgetown, Ontario). Standard lipids were obtained from Avanti Polar Lipids (Alabaster, Alabama).

Preparation of modified OCT: OCT contains a benzalkonium salt as a preservative, which can suppress the ionization of analytes during the MALDI process. Therefore, a modified OCT embedding agent was used that supported the placental tissue during cryostat sectioning and caused minimal interference in the MALDI-MS acquisition. A 10% (w/v) PVA 6-98 solution made in HBSS was microwaved for 30 seconds and then shaken for 16 cycles in order to dissolve the PVA 6-98. After cooling to room temperature, PPG 2000 and sodium azide were added to make the final modified OCT (mOCT) solution: 10% (w/v) PVA 6-98, 8% (v/v) PPG 2000, 0.1% (w/v) NaN3 [7].

Preparation of placental tissue sections: Preeclamptic (PE) and age-matched control (PTC) placental specimens were prepared as previously described [8]. Briefly, specimens were embedded in mOCT and then placed in a -20°C freezer. The placental tissue block was mounted onto the specimen disc of a cryostat (Leica Microsystems, Richmond Hill, ON) using mOCT. The tissue sections were sliced at a thickness of 12 μm at -25°C and mounted onto indium tin oxide (ITO)-coated glass slides. A thin matrix layer was applied to the tissue sections using an automated MALDI plate matrix deposition system (TM-Sprayer™, Leap Technologies, Carrboro, NC). A total of 5 mL of DHB solution (15 mg/mL in 50% acetonitrile/0.1% trifluoroacetic acid) was sprayed per slide during 4 passes at 140°C with a velocity of 400 mm/minute and a line spacing of 3 mm.

MALDI Imaging mass spectrometry: A time-of-flight tandem mass spectrometer (Sciex TOF/TOF 5800 System; Sciex, Concord, ON) was used to acquire the images. MALDI mass spectra were obtained using a Nd:YAG laser (349 nm) at 3 ns pulse width and 400 Hz firing rate. To install the ITO-coated glass slides in the ionization chamber, we used a special holder having concavities. All data were acquired in the positive-ion reflector mode using an external calibration method. The external calibration lipids (Avanti Polar Lipids, Alabaster, AL) were deposited on the ITO-coated slides to minimize mass shift. In the imaging experiment, a total of 200 laser shots per point were irradiated (1 s/point) and the interval between data points were of 50 μm. The images were visualized using TissueView software (Sciex, Concord, ON). After MALDI imaging was completed, the glass coverslip containing the placenta section was carefully removed from the MALDI plate, dipped in methanol to remove the matrix and fixed prior to standard H&E staining or immunohistochemistry.

Western Blot Analysis

Western blotting was performed using tissue lysates and gradient fractions from TC, PTC and PE placentae. Primary antibodies were used in the dilutions stated in supplementary materials. Following incubation with horseradish peroxidase (HRP)-conjugated secondary antibodies, membrane blots were visualized using enhanced chemiluminescence (PerkinElmer Inc., Waltham, MA) and imaged on X-ray film (GE Healthcare, Mississauga, ON). For quantification purposes, bands of interest were digitized for analysis using a CanoScanLiDE20 image scanner (Canon Canada Inc. Mississauga, ON). Quantification of Western blots was accomplished by densitometry using Quantity One software (Biorad, Mississauga, ON). Densitometry of the bands was performed in the linear range of detection

Antibodies and HRP Substrates

Antibodies against caveolin-1 (N-20, rabbit [WB 1:1000]), Flottilin-2 (A-3, mouse monoclonal [WB 1:500]), ALK1 (C-20, goat [WB 1:100]), ALK5 (TGFβ RI R-20, rabbit [WB 1:200]), TβRII (C-16, rabbit [WB 1:100]) and ENG (H-300, rabbit [WB 1:1000; IP 1:100]) were purchased from Santa Cruz Biotechnology (Santa Cruz, CA). Goat polyclonal anti-rabbit CD63, CD9, HSP70, CD81 [WB 1:1000] were purchased from System Biosciences (Mountain View, CA). Goat polyclonal anti-FLT-1 was purchased from Invitrogen (Life Technologies Inc., Burlington, ON). Mouse monoclonal anti-Calnexin [WB 1:1000] and anti-MMP14 [WB 1:100; IP 1:10] were obtained from EMD Millipore (Billerica, MA). Mouse monoclonal anti-VE Caderin/CD144 [WB 1:1000] was obtained from Abcam (Cambridge, UK). Biotin-conjugated cholera toxin B subunit and anti-hTFR were purchased from Sigma–Aldrich (St. Louis, MO). Biotin-conjugated mouse anti-human PLAP was purchased from eBioscience (San Diego, CA). Mouse monoclonal P4A4 against ENG was a generous gift of Dr. M. Letarte (Hospital for Sick Children, Toronto). Secondary antibodies were HRP-conjugated goat anti-rabbit, HRP-conjugated goat anti-mouse IgG (Jackson ImmunoResearch Laboratories, West Grove, PA). HRP-conjugated streptavidin (Jackson ImmunoResearch Laboratories) was used to detect biotin labelled CTB.

Results

Placental Lipid Microdomain Characterization

After sucrose density centrifugation, 8 fractions were collected and analyzed for cholesterol and GM1, characteristic lipid markers of detergent-resistant membrane microdomains.[9] Both cholesterol and GM1 were detected in the low density fractions 1-4 (Supplementary Fig. 1 A-C). For subsequent analysis the fractions were pooled as follow: fractions 1–2 (A) corresponding to 10-15% sucrose density, fractions 3-4 (B) to 20%-25% sucrose density, fractions 5-6 (C) to 30%-35% sucrose and fractions 7-8 (D) to 40% sucrose density. Protein quantification of the pooled fractions revealed that the insoluble fractions A+B contained ~2% of total protein, while the soluble fraction C contained 15%, and fraction D the rest. Subsequently, the insoluble (A-B) and soluble (C-D) fractions from PE, PTC and TC placentae were further analyzed. Equivalent amounts of protein of the four fractions (A-D) were subjected to SDS–PAGE, transferred to nitrocellulose membrane and immunoblotted for markers of caveolae and lipid rafts (caveolin-1 and flottilin-2, respectively) as well as for a marker of detergent-soluble fractions (calnexin)[10,11]. Insoluble fractions (A and B) were enriched in both lipid microdomain markers whereas soluble fractions (C and D) were immunopositive for calnexin (Supplementary Fig. 1D) and TFRC (Fig. 3C). The fractions were also assessed for placental alkaline phosphatase (PLAP) activity. PLAP is a well-known epithelial lipid raft marker present in the apical syncytiotrophoblast microvillous membrane [10,12]. The enrichment of PLAP activity in the DRM-containing fractions (A and B) relative to the activity in the initial whole tissue homogenates (Supplementary Fig. 1E) indicate that the insoluble fractions isolated from whole placentae contain a significant amount of apical (syncytial) membrane microdomains. No significant differences in PLAP enrichment between

PE *vs* PTC DRMs was observed. Saponin is known for its ability to disorganize the plasma membrane and to promote a shift in protein localization [13]. To further validate the DRM isolation method, placental homogenates were treated with Triton X-100 in the presence and absence of 1% saponin and membranes were then fractionated on a sucrose gradient. As anticipated, treatment with saponin decreased the cholesterol amount in the DRMs (insoluble fractions A,B; Supplementary Fig. 1F) that was accompanied by a shift of GM1 and caveolin-1 from the insoluble fractions to the detergent-soluble fractions (C and D) (Supplementary Fig. 1G).

Supplementary Table 1: Sphingomyelin content from detergent resistant fractions of total (**A**) and apical (**B**) membranes from preeclamptic (PE), preterm (PTC) and term control (TC) placentae**.**

A: Total placental membranes

SM Species	TC	PTC	PE
	ng/µg cholesterol		
**SM 16:0**	287.09 ± 42.54	361.08 ± 91.04	302.79 ± 31.73
**SM 18:0**	24.95 ± 3.09	41.06 ± 6.97	72.31 ± 9.13 *
**SM 18:1**	3.57 ± 0.31	8.00 ± 1.97	5.96 ± 1.46
**SM 24:0**	125.08 ± 24.77	264.68 ± 55.98	174.53 ± 26.25
**SM 24:1**	7.32 ± 0.74	110.45 ± 24.69	92.35 ± 32.39

Data are expressed as mean ± s.e.m. (TC: n=4; PTC: n=6; PE; n=10 different placentae). *P<0.05 by one-way ANOVA and posthoc Dunnet test.

B: Apical placental membranes

SM Species	TC	PTC	PE
	ng/µg cholesterol		
**SM 16:0**	245.72 ± 33.80	208.61 ± 21.94	331.32 ± 14.85
**SM 18:0**	33.84 ± 2.20	26.98 ± 4.33	61.87 ± 6.54 *
**SM 18:1**	9.27 ± 2.55	4.05 ± 0.65	14.86 ± 2.17
**SM 24:0**	117.57 ± 15.87	127.86 ± 10.88	169.22 ± 7.03
**SM 24:1**	51.26 ± 5.37	75.84 ± 9.85	52.26 ± 6.84

Data are expressed as mean ± s.e.m. (TC: n=6; PTC: n=3; PE; n=3 different placentae). *P<0.05 by one-way ANOVA and posthoc Dunnet test.

Supplementary Table 2: Sphingomyelin content in Golgi stacks isolated from preeclamptic (PE) and term (TC) placentae

SM species	TC	PE
	(ng/µg proteins)	
**SM 16:0**	3.10±0.12	4.93±0.86
**SM 18:0**	0.58±0.10	1.62±0.37*
**SM 18:1**	0.07±0.01	0.15±0.04
**SM 24:0**	1.65±0.67	2.53±0.24
**SM 24:1**	0.72±0.32	1.29±0.18

Data are expressed as mean ± s.e.m. (n=3 different placentae per group). *P<0.05 by unpaired t-test.

Supplementary Legends

Supplementary Figure 1. Placenta lipid microdomain characterization. Distribution of lipid microdomain markers cholesterol (A) and GM1 (B) in isolated sucrose gradient fractions. Experiments were repeated twice with similar results using different placentae. (C) Cholesterol quantification (mean ± s.e.m.) by LC-MS/MS of detergent insoluble (A,B) and soluble (C,D) membrane fractions from PTC and PE placentae (n=6 per group). (D) Distribution of caveolin-1, flottilin-2, calnexin (repeated twice with similar results using different placentae), and (E) placental alkaline phosphatase (PLAP) activity (mean ± s.e.m., n=6 placentae per group) in detergent insoluble (A,B) and soluble (C,D) fractions from PTC and PE placentae. (F,G) Cholesterol quantification (F) and distribution of GM1 and caveolin-1 (G) in PTC and PE sucrose gradient fractions after saponin treatment. Cholesterol data are presented as mean ± s.e.m. (n=3 placentae per group). GM1 and caveolin-1 was repeated two times with similar results using different placentae.

Supplementary Figure 2. (**A**) Quantification of sphingomyelin in lipid rafts of apical (syncytial) membranes from PTC (n=3) *vs* TC (n=3) placentae. (**B**). Distribution of ENG between detergent soluble and insoluble membrane fractions of PE and TC placenta. ENG distribution was assessed by immunoblotting after SDS-PAGE under non-reducing conditions. Experiment was repeated twice with similar results using different placentae. (**C**) Densitometry of sENG present in apical lipid rafts normalized by flottilin-2. Experiment was repeated twice with 3 placentae for every group. (**D**) Truncated ENG expression was determined by immunoprecipitation of the shortened isoform of ENG (sENG) from apical insoluble membranes of TC and PE placenta (n=3 different placentae per group) with P4A4 antibody followed by immunoblotting with H300 antibody. Standard (St): mix of recombinant full-length and truncated endoglin. (E) Representative

immunoblot of sFLT1 expression in the soluble and insoluble fractions of total TC and PE membranes. Experiment was repeated twice with similar results using different placentae. (F) Sphingomyelin changes (determined by LC-MS/MS) in anti-ENG immunoprecipitates of soluble membrane fractions of PE vs TC placenta. The SM alteration is expressed as Δ variation (PE-PTC/PTC*100) (PE, n=4; TC, n=4 different placentae). SM numbers indicate fatty acid chain length on D-erythro-sphingosylphosphorylcholine backbone. Data are presented as mean ± s.e.m.

Supplementary Figure 3. Interactions between truncated ENG, MMP14 and sphingomyelins in lipid rafts of apical (syncytial) membranes. (A) Interaction of HIS-conjugated peptide encompassing extracellular domain of ENG with various sphingomyelins as determined by protein overlay assay. Experiment has been repeated two times with similar results. (B) Sphingomyelin species in MMP14-precipitated DRMs from PE vs TC apical membranes. The alteration in SM is expressed as Δ variation (PE-TC/TC*100) (PE, n=4; TC, n=4 different placentae). Data are expressed as mean ± s.e.m. (C) Interaction between MMP14 and ENG in DRMs of apical microvillous membranes of TC and PE placentae (n=2 per group) as determined by co-immunoprecipitation assay.

Supplementary Figure 4. SM-18:0 content, MPP14 interaction with ENG and their association with SM-18:0 is increased in Golgi stacks of preeclamptic placenta. (A) Immunoblotting for Golgin-97 (Golgi marker) and pan-cadherin (marker of plasma cell membrane marker) of Golgi stacks isolated from preeclamptic placenta. (B) Changes in SM species (mean ± s.e.m) of isolated Golgi stacks from TC and PE placentae (n=3 different placentae per group). (C) Interaction between MMP14 and ENG in Golgi stacks of TC and PE placentae (n=2 per group) as determined by co-immunoprecipitation assays. (D,E) SM alterations (mean ± s.e.m.) in ENG

(D) and MMP14 (E) precipitated vesicles from PE and TC Golgi stacks. The SM changes are shown as D variation (PE-TC/TC*100). PE, n=3; TC, n=3 different placentae. (F) Quantification of CER 16:0 and 18:0 content in isolated Golgi fractions normalized to proteins (PE, n=3; TC, n =3 different placentae; *p < 0.05 by unpaired t-test). Data are presented as mean ± s.e.m.

Supplementary Figure 5. SM18:0 content of CD63-positive placental exosomes in maternal sera. LC-MS/MS quantification of SM-18:0 in CD63-precipitated exosomes from PE and TC maternal blood (PE, n=3; TC, n =3). Data are presented as mean ± s.e.m. Significance (*p<0.05) was determined by unpaired t-test.

Supplementary Figure 6. Full-length blots of Figure 2.

Supplementary Figure 7. Full-length blots of Figure 3.

Supplementary Figure 8. Full-length blots of Figure 4.

Supplementary Figure 9. Full-length blots of Figures 5 and 6.

Supplementary Figure 10. Full-length blots of Figure 7.

References

1 Caniggia, I., Taylor, C. V., Ritchie, J. W., Lye, S. J. & Letarte, M. Endoglin regulates trophoblast differentiation along the invasive pathway in human placental villous explants. *Endocrinology* **138**, 4977-4988, doi:10.1210/endo.138.11.5475 (1997).

2 Dowler, S., Kular, G. & Alessi, D. R. Protein lipid overlay assay. *Sci STKE* **2002**, pl6, doi:10.1126/stke.2002.129.pl6 (2002).

3 Taylor, R. S., Jones, S. M., Dahl, R. H., Nordeen, M. H. & Howell, K. E. Characterization of the Golgi complex cleared of proteins in transit and examination of calcium uptake activities. *Mol Biol Cell* **8**, 1911-1931 (1997).

4 Tibboel, J., Joza, S., Reiss, I., de Jongste, J. C. & Post, M. Amelioration of hyperoxia-induced lung injury using a sphingolipid-based intervention. *Eur Respir J* **42**, 776-784, doi:10.1183/09031936.00092212 (2013).

5 Bielawski, J., Szulc, Z. M., Hannun, Y. A. & Bielawska, A. Simultaneous quantitative analysis of bioactive sphingolipids by high-performance liquid chromatography-tandem mass spectrometry. *Methods* **39**, 82-91, doi:10.1016/j.ymeth.2006.05.004 (2006).

6 Yoo, H. H., Son, J. & Kim, D. H. Liquid chromatography-tandem mass spectrometric determination of ceramides and related lipid species in cellular extracts. *J Chromatogr B Analyt Technol Biomed Life Sci* **843**, 327-333, doi:10.1016/j.jchromb.2006.06.025 (2006).

7 Berry, K. A. *et al.* MALDI imaging MS of phospholipids in the mouse lung. *J Lipid Res* **52**, 1551-1560, doi:10.1194/jlr.M015750 (2011).

8 Melland-Smith, M. *et al.* Disruption of sphingolipid metabolism augments ceramide-induced autophagy in preeclampsia. *Autophagy* **11**, 653-669, doi:10.1080/15548627.2015.1034414 (2015).

9 Ermini, L. *et al.* Different glycoforms of the human GPI-anchored antigen CD52 associate differently with lipid microdomains in leukocytes and sperm membranes. *Biochem Biophys Res Commun* **338**, 1275-1283, doi:10.1016/j.bbrc.2005.10.082 (2005).

10 Godoy, V. & Riquelme, G. Distinct lipid rafts in subdomains from human placental apical syncytiotrophoblast membranes. *J Membr Biol* **224**, 21-31, doi:10.1007/s00232-008-9125-5 (2008).

11 Rashid-Doubell, F. *et al.* Caveolin-1 and lipid rafts in confluent BeWo trophoblasts: evidence for Rock-1 association with caveolin-1. *Placenta* **28**, 139-151, doi:10.1016/j.placenta.2005.12.005 (2007).

12 Riquelme, G. *et al.* Lipid rafts and cytoskeletal proteins in placental microvilli membranes from preeclamptic and IUGR pregnancies. *J Membr Biol* **241**, 127-140, doi:10.1007/s00232-011-9369-3 (2011).

13 Simons, K. & Toomre, D. Lipid rafts and signal transduction. *Nat Rev Mol Cell Biol* **1**, 31-39, doi:10.1038/35036052 (2000).

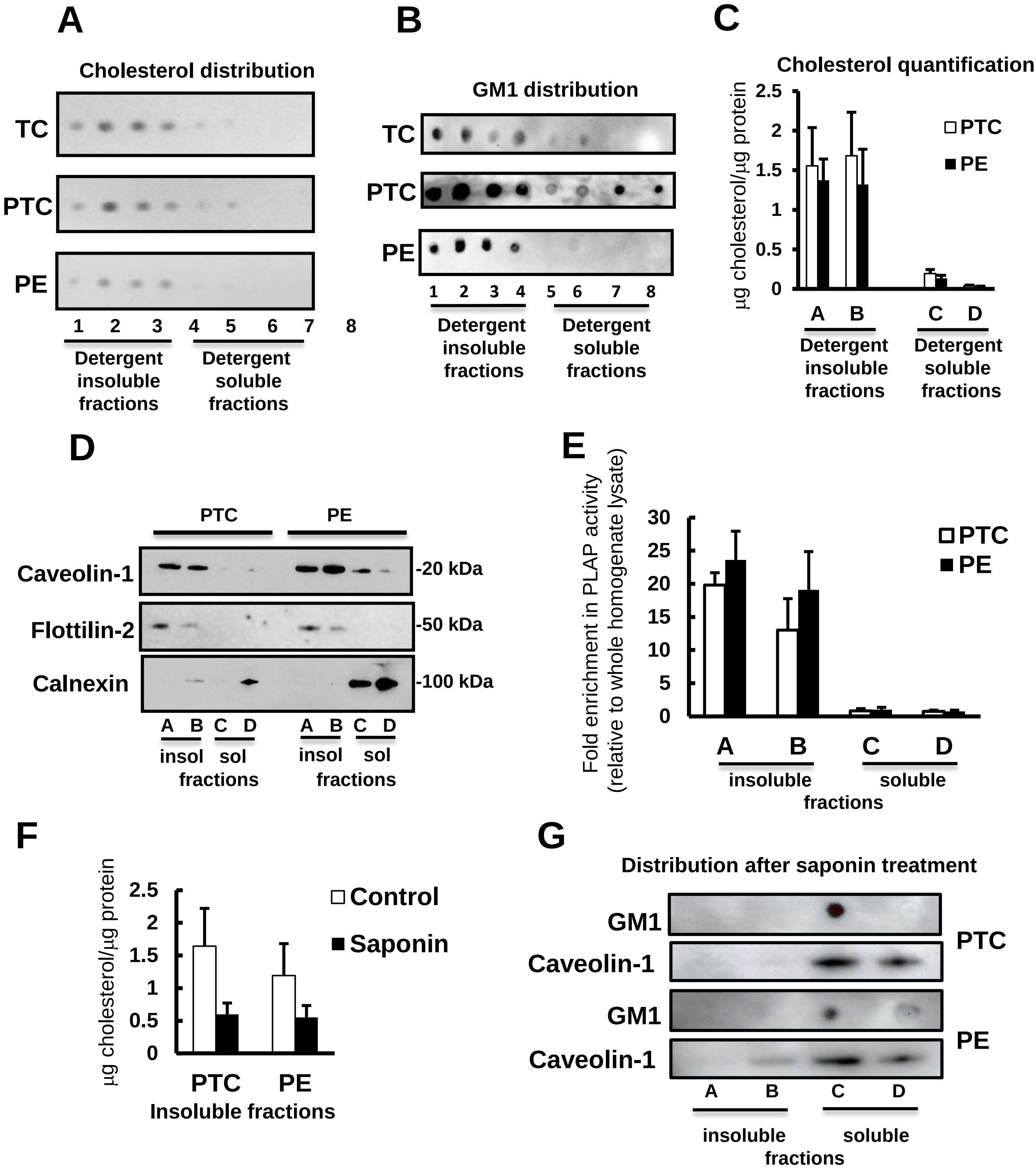

Supplementary Figure 1

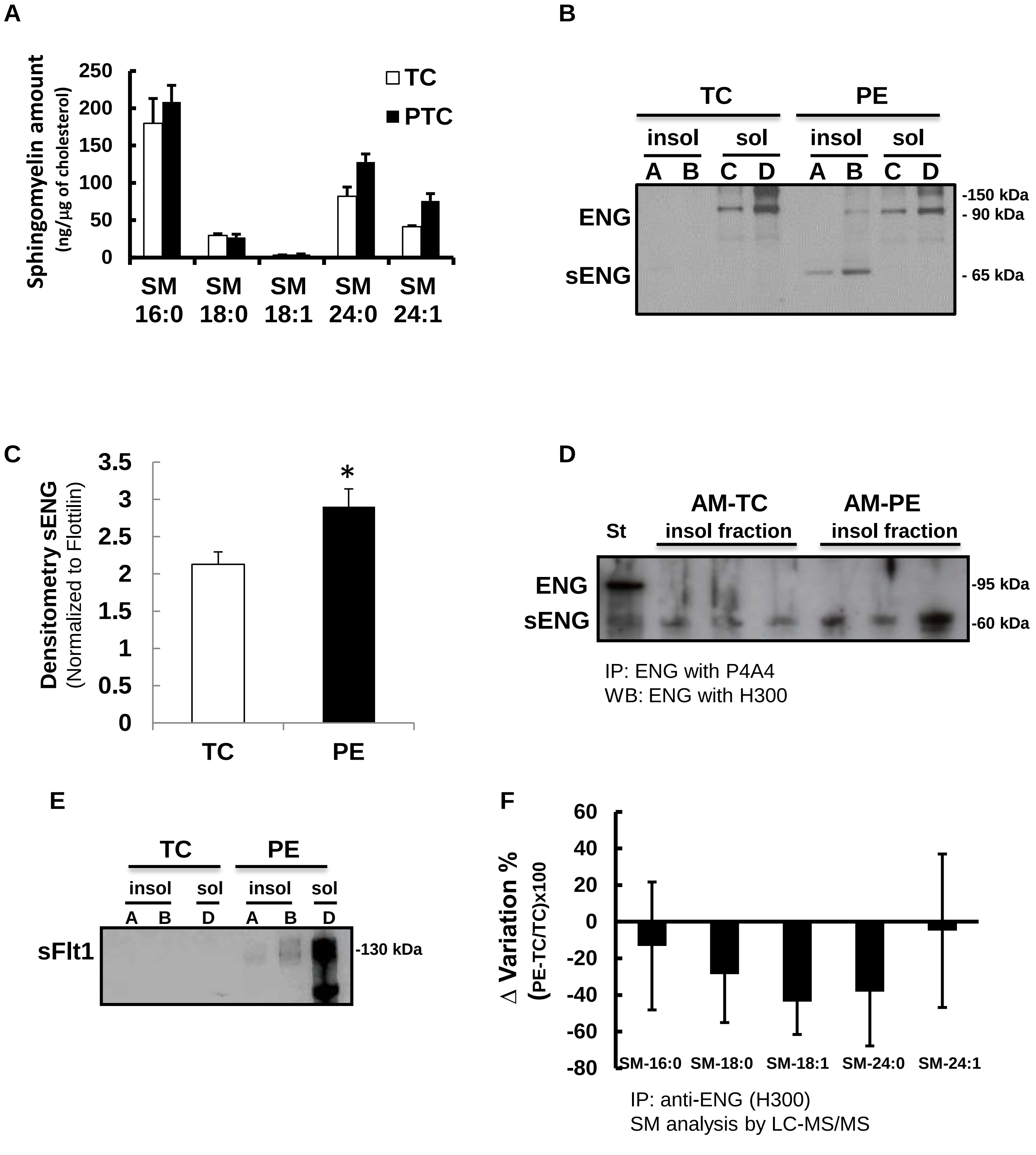

Supplementary Figure 2

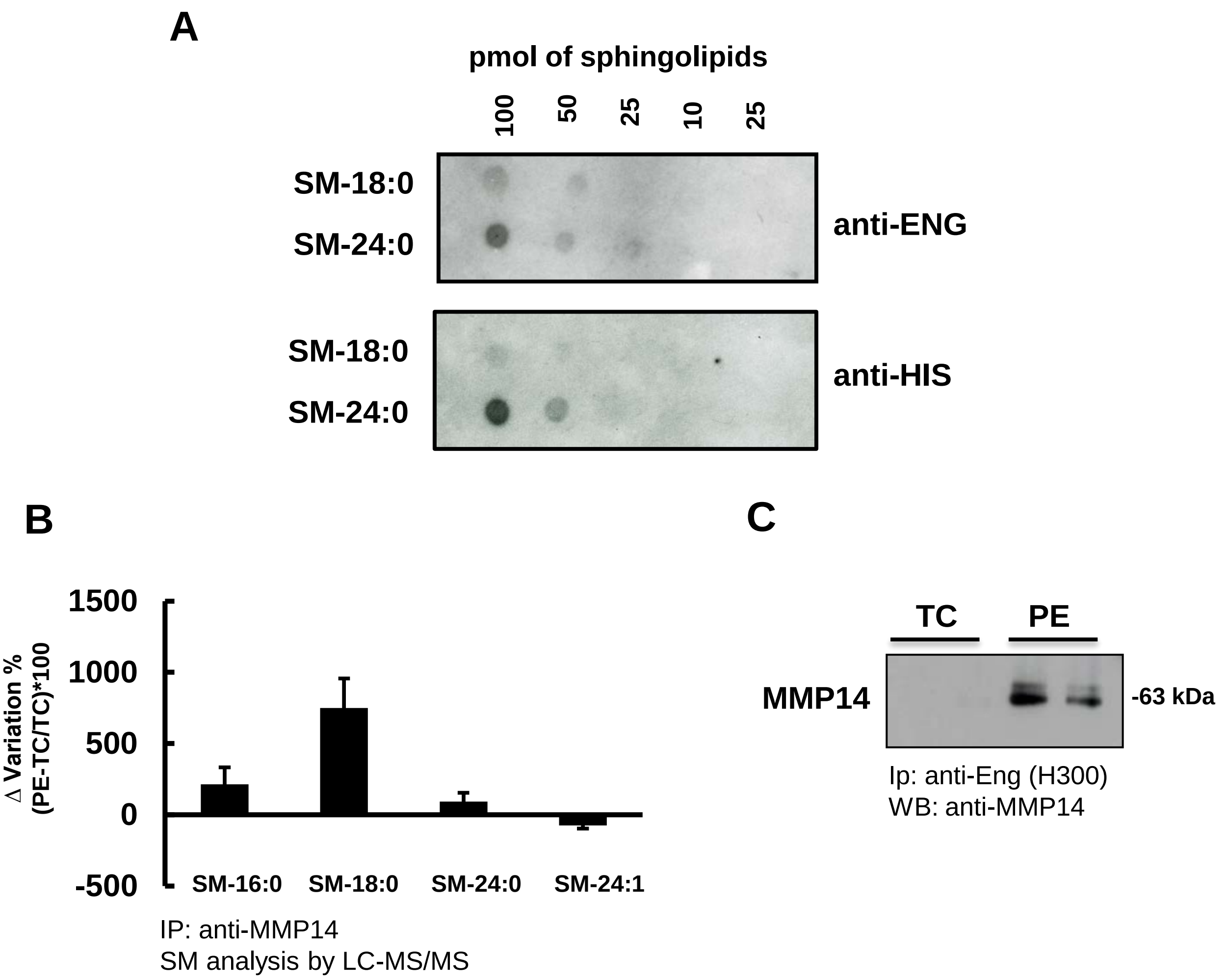

Supplementary Figure 3

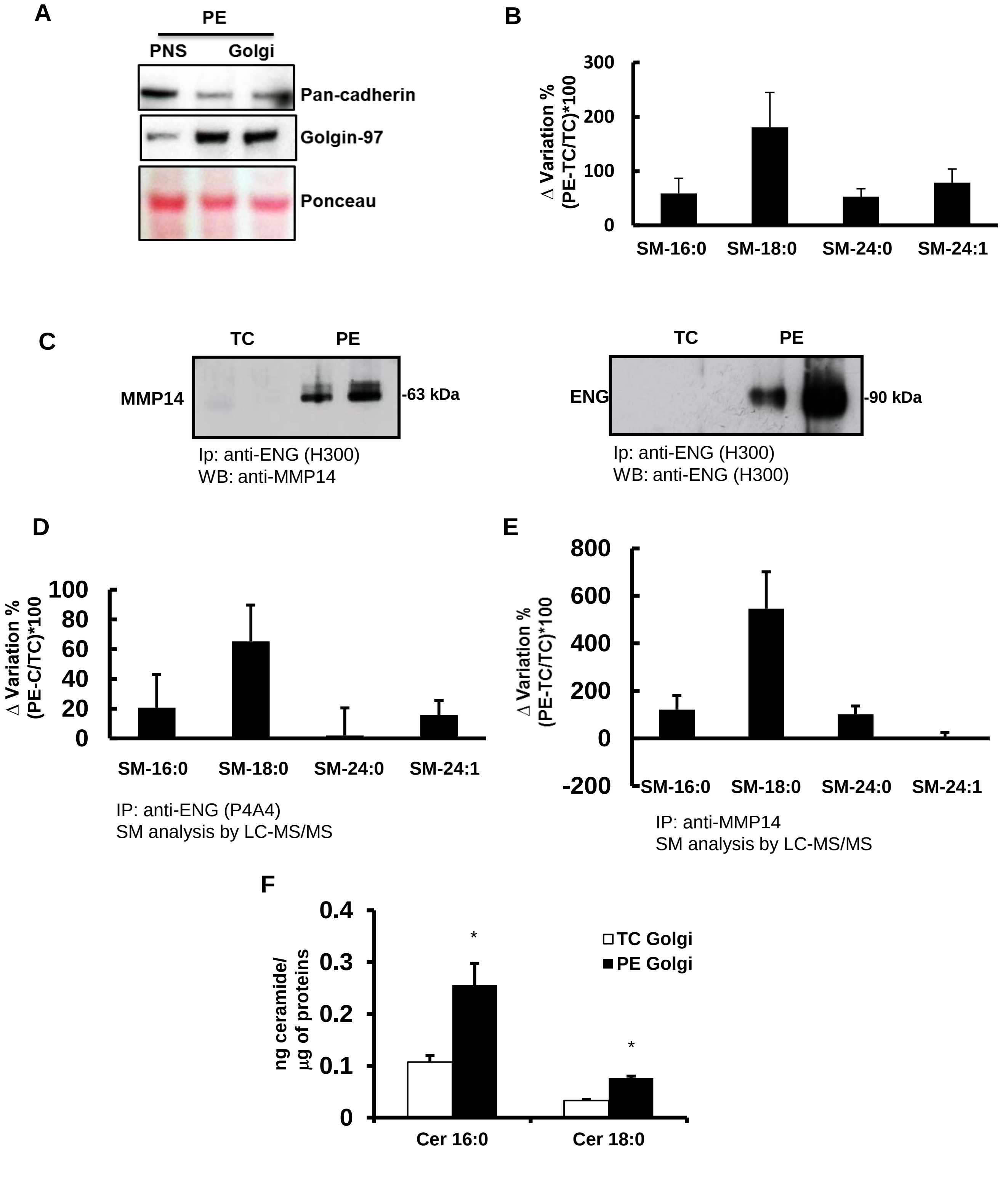

Supplementary Figure 4

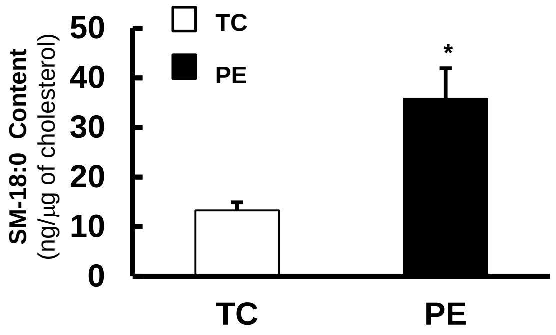

IP: anti-CD63
SM analysis by LC-MS/MS

Supplementary Figure 5

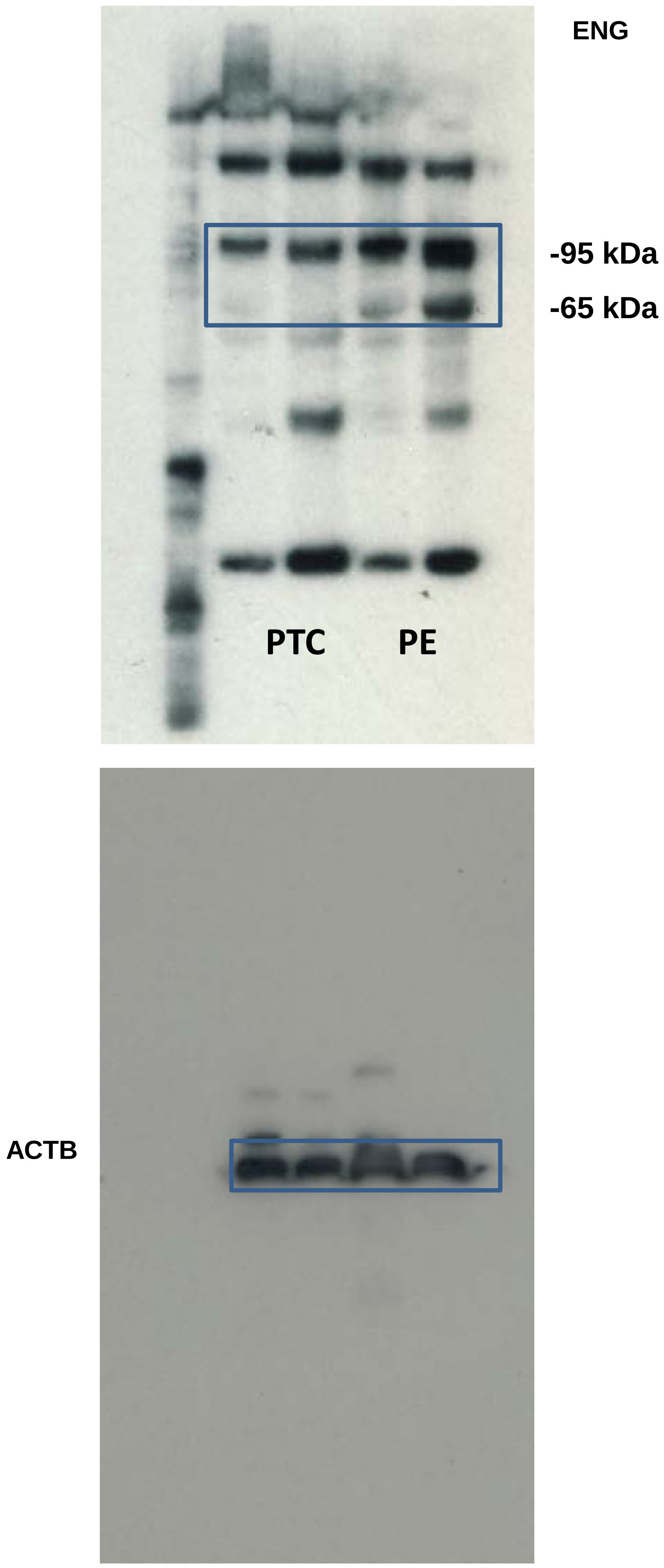

Supplementary Figure 6

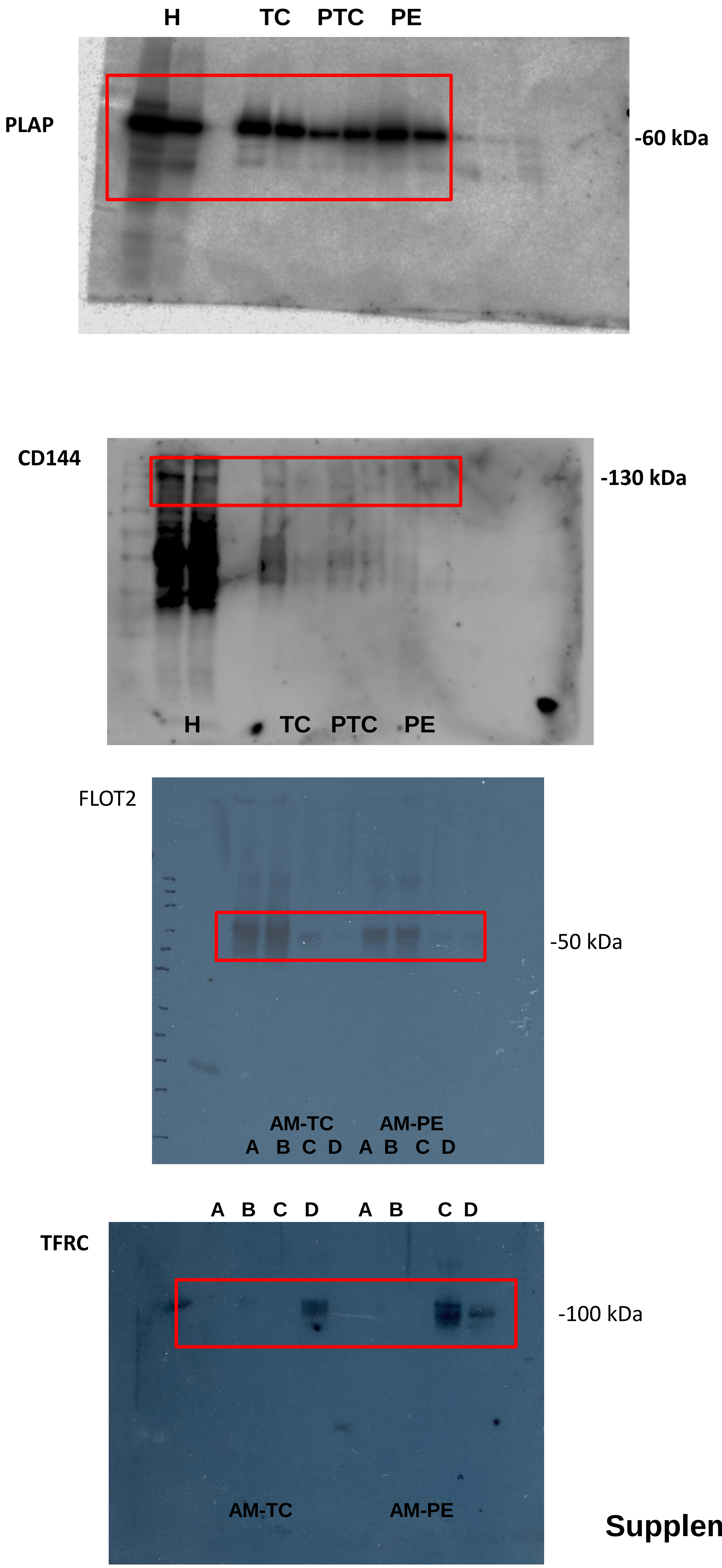

Supplementary Figure 7

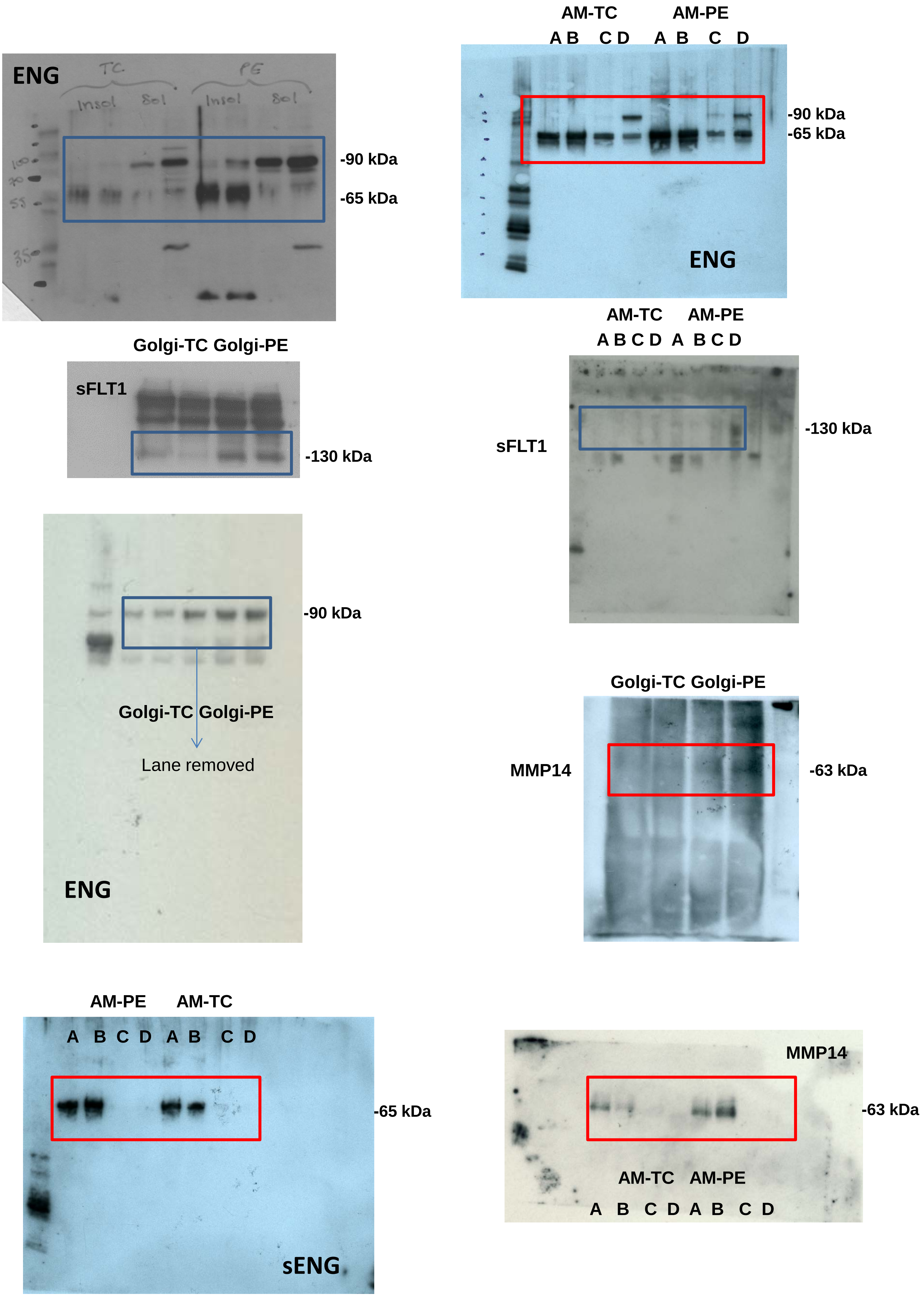

Supplementary Figure 8

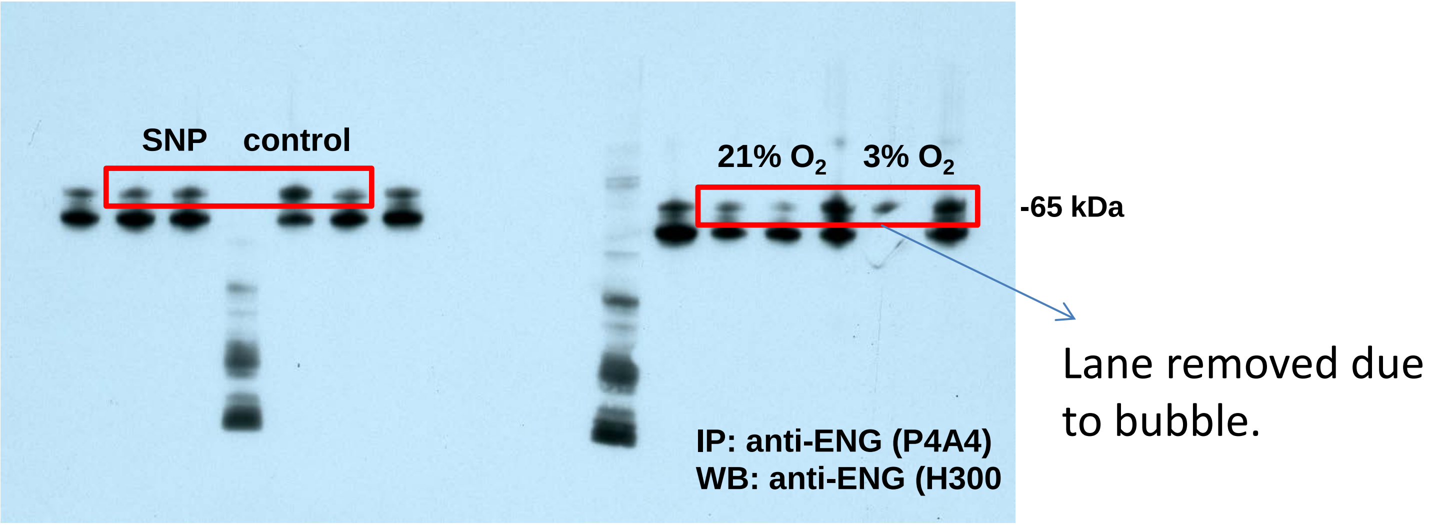

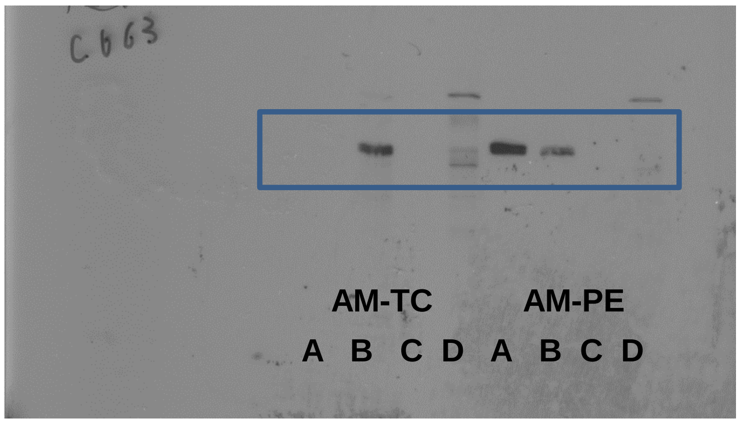

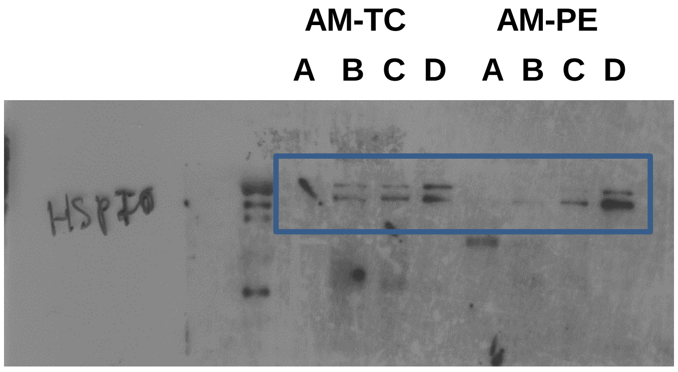

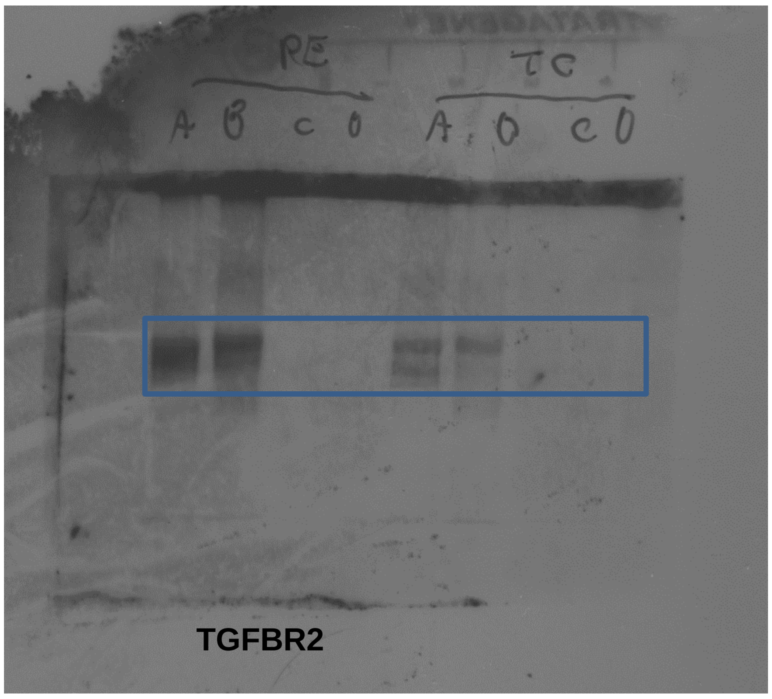

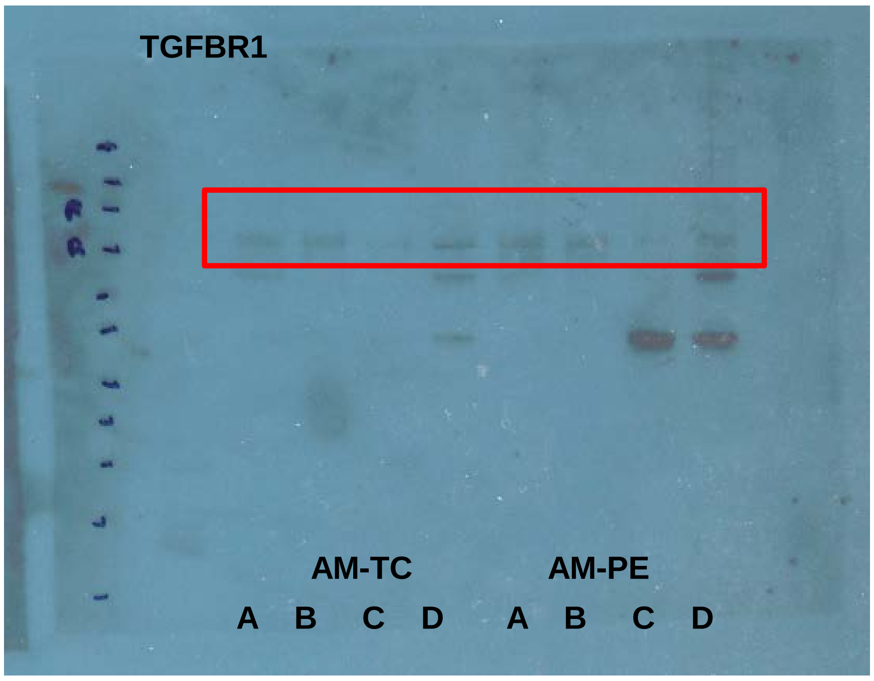

Supplementary Figure 9

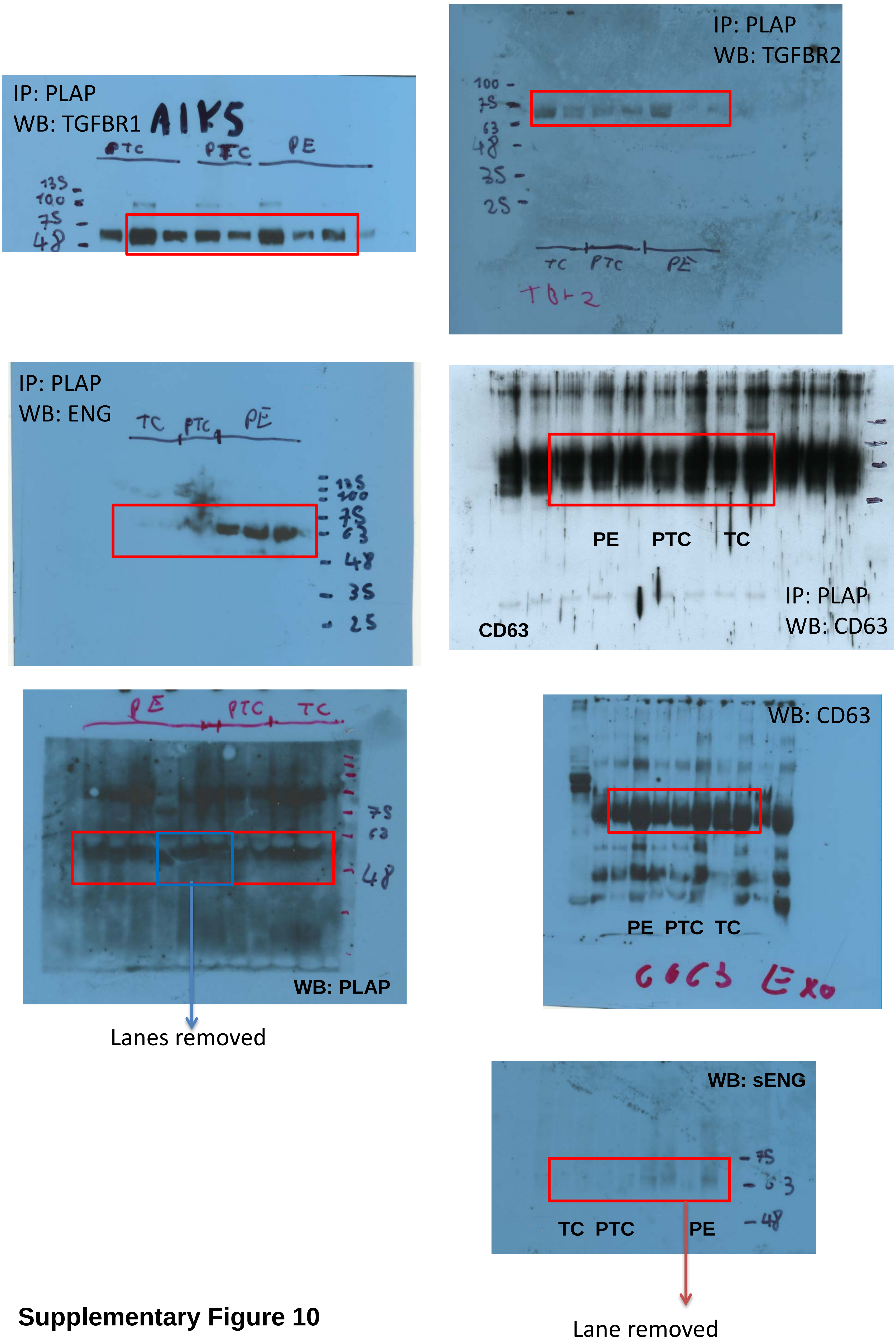

Supplementary Figure 10
